# From *α-*to *β*-diversity: Understanding the historical, present, and future diversity patterns of Fagaceae in Southwestern China

**DOI:** 10.1016/j.heliyon.2024.e41474

**Published:** 2024-12-31

**Authors:** Bikram Pandey, Fengying Zhang, Basu Dev Poudel, Rong Li, Mohammed A. Dakhil, Bishal Gurung, Zhaxi Luobu, Yawen Gan, Ziyan Liao, Lin Zhang

**Affiliations:** a CAS Key Laboratory of Mountain Ecological Restoration and Bio-resource Utilization and Ecological Restoration Biodiversity Conservation Key Laboratory of Sichuan Province, Chengdu Institute of Biology, Chinese Academy of Sciences, Chengdu, 610041, Sichuan, China; b CAS Key Laboratory for Plant Diversity and Biogeography of East Asia, Kunming Institute of Botany, Chinese Academy of Sciences, Kunming, 650201, Yunnan, China; c Sichuan Provincial Institute of Forestry and Grassland Inventory and Planning, Chengdu, 610081, Sichuan, China; d Environmental Services Nepal, Kathmandu, Nepal; e School of Ecology and Environment, Northwestern Polytechnical University, Xi'an, China; f Botany and Microbiology Department, Faculty of Science, Helwan University, Cairo, 11790, Egypt; g University of Chinese Academy of Sciences, Beijing, China; h Institute of Agriculture Research, Tibet Academy of Agriculture and Animal Husbandry Sciences, Lhasa, 850032, Tibet, China

**Keywords:** Beta-ratio, Geographic distance, Macro-diversity pattern, Species distribution modelling, Species turnover

## Abstract

Macroecological research aims to understand factors influencing species composition and diversity. Understanding the distribution patterns of species is essential for prioritizing areas for conservation. This study investigates the alpha (*α*) and beta (*β*) diversity facets of Fagaceae across past (historical), present, and future timelines in Southwestern China. We used over 11,000 geographical observations to predict the spatial patterns of the *α*- and *β*-diversity of 120 species. We modeled the *α*-diversity via stacking prediction using an individual species distribution model at 50 km × 50 km grid cells. We used Sørensen dissimilarity to quantify total *β*-diversity and its components - turnover (*β*_SIM_) and nestedness (*β*_NES_). We integrated climate variables along with topographic and plant trait predictors to understand the species diversity. Finally, simultaneous autoregression (SAR) model was used to evaluate the effects of predictor variables on the *α*- and *β*-diversity patterns. Our results indicate a projected decline in α-diversity and an increase in β-diversity in the future. The findings underscore that the species *β*_SIM_ is a driving factor of differing species composition during the past and present periods, while *β*_NES_ will be a dominant factor in the future. Similarly, climatic and topographic factors significantly influenced the *α-*diversity and the *β*-diversity. In the future, climatic variables will play a significant role in determining the diversity patterns. By closely studying how various species respond and adapt to these changes, we can gain valuable insights into the dynamics of ecosystems and the potential threats to biodiversity.

## Highlights

• This study addresses the challenge of understanding biodiversity pattern of Fagaceae in Southwestern China.
• Due to rapid climate change, it's crucial to identify ecologically significant areas for conservation.
• The study integrates ecological and biogeographical sites to determine the species diversities.
• We recommend targeting smaller, multiple sites for the conservation of Fagaceae at the present.
• However, a single large nature reserve should be suitable for its conservation in the future.

## Graphical abstract

**Figure:**
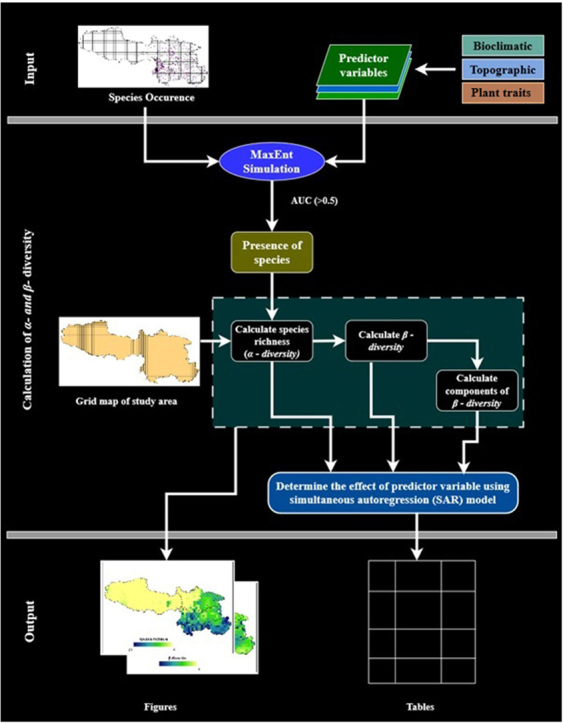

## 1 Introduction

Studying biodiversity patterns and their respective driving processes is invariably challenging. Understanding the geographic pattern and environmental drivers of biodiversity is the primary goal of ecologists and bio-geographers. These patterns, largely summarized into *α-*, *β-*, and γ-diversity components [1], are used to describe and understand patterns of species diversity across different scales [[2], [3], [4], [5]]. Among these, *α*-diversity and *β*-diversity emerge as pivotal aspects in diversity measurement [[6], [7], [8], [9]]. Rapid climate change is currently inducing significant alterations in climatic conditions, posing a global threat to species diversity [10]. Therefore, both components aid in identifying key areas for conservation [3,6,[10], [11], [12], [13]].

*α*-diversity refers to the diversity of species (species richness) in any ecological unit. It represents the species richness at a local scale and provides insights into the diversity within a particular community or ecosystem [14], while *β*-diversity is a key concept in community ecology and measures the dissimilarity among two or more communities [7]. The study of *β*-diversity entails analyzing the extent of the differences in the species composition across different locations, with the goal of revealing the fundamental mechanisms that give rise to these variations [7]. Both of these biodiversity patterns within a region may result from a recent increase in the rate of speciation, which has generated a cradle of biodiversity. It can be used to understand how communities change over time and in response to different environmental conditions [13,15,16]. In general, the *β*-diversity among species can serve as an indicator of how species respond to existing environmental conditions and/or geographical barriers [17]. Moreover, the total *β-*diversity can be partitioned into species turnover (*β*_SIM_) and nestedness (*β*_NES_) components [7]. Species turnover involves the transition of species from one site to another, leading to a reduced proportion of co-occurring species in both communities. Conversely, the nestedness of the species *β*-diversity pertains to the variance in the species richness between two communities where the less abundant community represents a more diverse subset of the richer community [6,12].

The predominant approach to characterizing regional biodiversity and biogeographical processes has been the taxonomic-based analysis of the community assemblage [18]. Although *α-* and *β*-diversity have been frequently studied at the taxonomic level [2,13,15,19,20], the increasing impacts of climate change have generated a pressing need to comprehensively comprehend the fluctuations within these indices. Moreover, the *β-*diversity, which pertains to the dissimilarities in the species assemblages or ecological communities among two or more locations or habitats, and can be used to assess the spatial scaling of biodiversity loss [10]. Therefore, these approaches have been proven to be particularly valuable in unraveling the complex relationships between climatic, and topographic factors and their distributions across geographic regions, shedding light on the intricate interplay between ecological and biogeographical dynamics [2,19,[21], [22], [23]].

Climate plays a significant role in shaping both species richness and beta diversity. Climatic factors such as temperature and precipitation have a direct impact on resource availability and habitat suitability. Climate gradients, such as temperature and precipitation variations, create habitat diversity across regions, leading to variations in species compositions [13,19,20,24]. Areas with consistent and favorable climates are more likely to support a higher species richness. This variation can result in distinct species compositions, contributing to a higher *β*-diversity [15,25]. Topographic heterogeneity is another key driver of plant species richness and *β*-diversity as it contributes to habitat variation. It determines resource availability, niche differentiation, ecological gradients, and species turnover. Conversely, assemblages in distinct environments isolated by geographic and historical barriers will result in a pattern of high *β-*diversity [13,15,20]. Thus, in areas with high topographic heterogeneity, distinct habitats and microclimates are more prevalent, leading to a higher species richness and turnover. Furthermore, plant traits, particularly height and seed volume, serve as proxies for both dispersal ability and competitiveness, helping to explain the extent of environmental filtering and dispersal [[26], [27], [28]]. Therefore, this study emphasizes the importance of incorporating climatic, topographic, and plant traits into conservation planning of Fagaceae in Southwestern China (hereafter to as SW China).

The focus of this study was to determine the spatial patterns of the *α-* and *β*-diversity of Fagaceae during the historical (Last Glacial Maximum, LGM), present and future across SW China. SW China is a crucial ecoregion and a biological hotspot [29]. This angiosperm family consists of eight extended genera and approximately 1000 species that are widespread throughout tropical and temperate regions, mostly in the Northern hemisphere [30,31]. China is home to 294 species that are grouped into six genera and mostly found in SW China [31]. The family Fagaceae is diverse in SW China with around 206 species, commonly found in various types of evergreen broadleaf forests across the floristic region. There are 163 endemic species of Fagaceae in China, of which approximately 72 are found in SW China [32].

Fagaceae species have economic importance as a source of timber, fuelwood, and food [29]. The presence of several dominant communities and keystone genera of Fagaceae in SW China (eg- *Quercus, Castanae,* and *Lithocarpus*), plays a critical role in forest ecosystem. The family has a relatively wide distribution and contribute significantly to the structure and function of both tropical and temperate forests in SW China [33]. This broad distribution makes it a suitable to understand its distribution dynamics at the regional scale.

To gain insights into the distribution of Fagaceae across SW China, we pursued several key objectives: (i) to model and describe the *α*- and *β*-diversity of Fagaceae across three distinct time periods: LGM, the present period, and the future. By examining the diversity patterns at different points in time, we sought to uncover the variations in the species richness at regional scales (*α*-diversity) and the turnover and dissimilarity in the species composition between different locations (*β*-diversity), (ii) to investigate the impact of climate, topographic, and trait variables on the observed diversity patterns. Understanding these drivers is crucial for elucidating the ecological processes that govern the distribution of Fagaceae in the area, and (iii) the relative contribution of the *β-*diversity and its specific components, namely, the turnover (*β*_SIM_) and nestedness (*β*_NES_), as well as the beta-ratio (*β*_ratio_), in determining the distribution of Fagaceae in SW China. By discerning the importance of each component, we aimed to shed light on the underlying ecological mechanisms that govern the dissimilarity and species turnover in different locations. Thus, the result of this study provides valuable insights into the spatial distribution and ecological dynamics of Fagaceae in the SW China and hence a reference for spatial conservation prioritization.

## 2 Material and methods

### 2.1 Study area, distribution data, and spatial differentiation

The present study area is primarily delimited by the boundaries of the southwestern region of China and covers approximately 2.854 million km^2^ (Supplementary Fig. 1.1). Due to the large geographical area, the region is characterized by diverse plant species and topographic, edaphic, and climatic variations. Therefore, we created an enhanced database comprised of 221 species of Fagaceae in SW China. This database was constructed using observation records acquired from various sources, including the National Specimen Information Infrastructure (NSII, http://www.nsii.org.cn/; accessed between September 2022 and February 2023), Chinese Virtual Herbarium (CVH, http://www.cvh.ac.cn/; accessed between September 2022 and February 2023), and Global Biodiversity Information Facility [34]. Among these species, 101 had fewer than 10 georeferenced records, leading to their exclusion from the subsequent analysis. Further, Catalogue of Life (https://www.catalogueoflife.org/; accessed between September 2022 and February 2023) was used to validate the name of species for nomenclature and synonyms. To ensure the data quality, we carefully eliminated duplicate records and occurrences with incomplete coordinates. Consequently, our analysis focused on a subset of 120 species, resulting in a total collection of over 11,000 records for use in this study.

As part of our methodology, we partitioned the entire study area into 1095 grid cells, each measuring 50 × 50 km. Subsequently, the distribution maps of 120 target species were overlaid onto these grid cells. Following the method of Liao et al. [15], to ensure robust analysis, we excluded grid cells with less than 75 % land area coverage and those with no recorded species (Supplementary Fig. 2). This stringent selection process allowed us to focus on areas with sufficient data coverage, enabling a comprehensive and reliable examination of the species distribution patterns within the study area.

### 2.2 Predictor variables

To characterize the predictor variables that may drive the patterns of the historical, present, and future *α-* and *β*-diversity, and to identify the factors driving these patterns, we used bioclimatic, topographic data and plant traits in our evaluation.

In the Late Quaternary, the global climate has significantly influenced the development of various macroecological patterns [35]. Nineteen paleoclimatic and three paleo-topographic variables were used to evaluate the *α-* and *β-*diversity of Fagaceae in SW China (Supplementary Table 1.1). In this study, we utilized downscaled paleoclimatological and paleo-digital elevation model (paleoDEM) data from the LGM, approximately 21,000 years ago. These data were obtained from climatologies at high resolution for Earth's land surface areas (CHELSA) V1.2, which has a 30-arcsecond resolution [36]. The CHELSA LGM data are based on the implementation of the CHELSA algorithm on the Paleoclimate Modelling Intercomparison Project 3 (PMIP3) Community Climate System Model version 4 (CCSM4) simulation.

The present period dataset is comprised of 19 bioclimatic, three topographic variables, and four life history plant traits. A dataset containing nineteen bioclimatic variables was obtained from the WorldClim repository with a spatial resolution of 30 arcseconds (ver. 2.1), representing the current climatic conditions [37]. A present-day digital elevation model (DEM) was used to calculate the mean elevation and elevation range, that represent topographic heterogeneity [19]. The DEM was downloaded from the National Aeronautics and Space Administration's (NASA) Shuttle Radar Topography Mission (SRTM) database with a 30-arcsecond resolution [38]. The geographical distance between each pair of grid cells was measured as the Euclidean distance between the mid-points of the grid cells. The life history plant trait includes – average tree height, leaf length, leaf width, and seed volumes. All these variables were derived from Chen et al., 2022 [27], Chen et al., 2023 [28], and Wu et al., 1999 [29].

The future dataset is comprised of 19 bioclimatic and three topographic variables. In accordance with the Intergovernmental Panel on Climate Change's (IPCC) 2050 target, we used climate change scenarios spanning from 2040 to 2060, which were obtained from the General Circulation Model (BCC-CSM2-MR), to simulate future environmental conditions [39]. To obtain diverse future predictions, we selected four distinct shared socioeconomic pathways: SSP-1.26, SSP-2.45, SSP-3.70, and SSP-5.85. These datasets had a resolution of 30-arcseconds were obtained from the World Climate Change Program (WCRP) Coupled Model Intercomparison Project six (CMIP6) through WorldClim.

We projected all of these variables from their respective resolutions to a 50 × 50 km grid covering our study area using ArcGIS (V 10.7.1).

### 2.3 Diversity measurement and statistical analyses

To address our first objective, we computed the historical, current, and future taxonomic diversity of Fagaceae and visualized the *α-* and *β*-diversity separately. We utilized range estimates for Fagaceae species, acquired from polygons and species distribution modeling, to create species assemblages within the designated grid cells. We measured the species richness as a component of the *α-*diversity, representing the number of different species within a 50 × 50 km grid cell. In general, the data on the geographical occurrence across the spatial scale were used to estimate the *α-* and *β-*diversity; however, this approach may be biased considering the objectives of this study. Therefore, the historical, current, and future species richness were estimated by stacking individual species distribution models using the maximum entropy approach (MaxEnt) [40]. To achieve this, MaxEnt model with the current species occurrence data and predictor variables as the inputs, generating a model that predicts the potential distribution of the species in present period. Similarly, we ensured that the potential species distribution for each time period was estimated based on the same present-day species distribution patterns but under different climatic and topographic scenarios reflective of the study area's historical, and future environments. The model built by MaxEnt can be used to project species distributions across different time periods (historical, present, future) by applying the same model to environmental conditions representative of those time periods. The MaxEnt model have advantage as it use presence only data, effectively handles both continuous and categorical environmental variables and delivers strong accuracy and predictive power [40]. To test and evaluate the quality of the model, we used 70 % of the occurrence data for training and the remaining 30 % for validation [24,41]. The jack-knife test was used to determine the contribution of each variable, while Pearson's correlation coefficient was applied to assess the correlation between variables and reduce multicollinearity in the analysis. Variables with correlation coefficients of r ≥ |0.7| were excluded from the initial environmental dataset [24] (Supplementary Tables (2.1–2.6)).

In the MaxEnt model, the AUC (Area Under the Curve) of the ROC curve is a widely used, threshold-independent metric that serves as the default criterion for assessing model accuracy [42]. To determine suitable areas for the species, we set the threshold to >0.5; in other words, only areas with a predicted habitat suitability probability of greater than 0.5 were considered suitable [43]. Subsequently, we counted the number of unique species occurrences within these suitable areas to calculate the species richness. We considered a species to be present in a grid cell when the value exceeded 0.5. The species richness was calculated as the total number of different species within an individual grid cell. We performed this analysis in ArcGIS.

To analyze the *β-*diversity, we employed the pairwise Sørensen dissimilarity index (*β*_SOR_) to assess the taxonomic *β*-diversity of Fagaceae across different time periods: historical, present, and future periods. Following Baselga [7], we further divided the *β*_SOR_ into two components: the Simpson dissimilarity index (*β*_SIM_) and the nestedness index (*β*_NES_). In this study, we computed three measures of the *β-*diversity: the Sørensen dissimilarity index (*β*_SOR_), the Simpson dissimilarity index for species turnover (*β*_SIM_), and the nestedness index (*β*_NES_). To determine the *β*-diversity components for each cell, we calculated the average values between the focal cell and its eight adjacent cells [44]. Additionally, following Dobrovolski et al. [22], we obtained the *β*_ratio_, which represents the ratio of *β*_NES_ to *β*_SOR_. This allowed us to compare the relative contributions of the nestedness and turnover components to the overall *β-*diversity between different sites. The value of the *β*_ratio_ range between 0 and 1. A value of >0.5 indicates that *β*_NES_ is a dominant factor, and species assemblages in species-poor sites largely consist of subsets found in species-rich sites. Conversely, a value of <0.5 suggests that *β*_SIM_ (species replacement/turnover) plays a more substantial role in driving the differences in the species compositions of sites. We used the customized scripts of *betapart* [45] i.e. “betagrid” function in R to calculate the *β-*diversity and its components [46].

First, we performed ordinary least squares (OLS) regression to determine the effect of predictors on the diversity patterns. The residuals of the OLS multi-predictor regression models for each diversity metric exhibited significant spatial autocorrelation, as detected through the Moran's I test. Such a spatial autocorrelation can lead to biased parameter estimates and incorrect error probabilities. To address this issue, spatial regression techniques such as spatial autoregressive models should be considered as they account for spatial dependencies. Ensuring accurate and reliable regression results in spatial data analysis is crucial. Therefore, to explore the relative impact of historical, current, and future predictors on the diversity patterns and address our second and third objectives, we employed a simultaneous autoregression (SAR) model [47]. Prior to applying the SAR, we conducted Pearson correlation tests between the predictor variables and omitted the highly correlated variables (r > 0.7) due to their multi-collinearity. The list of predictor variables used in analysis is mentioned in Supplementary Table 1.2.

We followed a procedure similar to that adopted by Liao et al. [15]. Finally, we selected the model with the highest Pseudo-*R*^2^ and lowest Akaike information criterion (AIC) values. The SAR models were implemented using the “spdep” package [48]. All of the statistical analyses were performed in R 4.2.0 [49], and the maps were generated in ArcGIS (v 10.7.1). A detail flowchart showing spatial modelling and statistical analyses is presented in Supplementary Fig. 1.2.

Additionally, the difference in the species richness (ΔSR) between the present and future were calculated. The ΔSR provides valuable insights into the diversity and composition of species over time, aiding in the identification of trends in the biodiversity [50]. Positive values indicate an increase in the species richness in a specific plot over time, while negative values indicate a decrease in the species richness.

## 3 Results

### 3.1 Geographic patterns of *α*-diversity

**Fig. 1:**
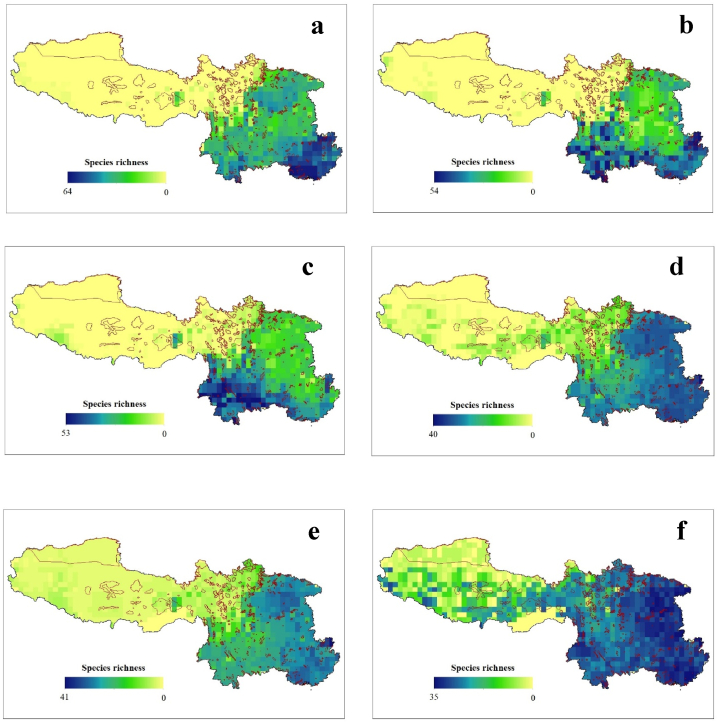
Geographic pattern of taxonomic species richness (*α*-diversity) of Fagaceae in Southwestern China during the historical (LGM) (a), present (b) and future (c–f) under climate change scenario- SSP1-2.6 (c), SSP2-4.5 (d), SSP3-7.0 (e), and SSP5-8.5 (f). The red boundary line represents the protected areas.

Based on our findings, we predict that of total 120 studied species, the number of Fagaceae species in SW China will gradually decrease in the future (Fig. 1, Supplementary Table 1.3). We observed no change in the number of species between the LGM, current, and SSP1-2.6 future scenarios. However, under climate change scenarios SSP2-4.5, SSP3-7.0, and SSP5-8.5, 63, 66, and 71 species, respectively, are projected to lose their habitats (Fig. 1d–f, Supplementary Table 2). Despite this decline in the species count, we found that the area of the species occurrence is expected to increase. During the LGM period, Fagaceae species occupied 599 grid cells in SW China (Fig. 1a). This number decreased to 538 in the present period (Fig. 1b). However, our predictions indicate that in the future, the number of grid cells occupied by Fagaceae will range from 681 to 1043 under the various climate change scenarios (Fig. 1c–f, Supplementary Table 2). During the LGM period, a grid cell contained the highest number of Fagaceae species (n = 64). In contrast, the least number of species is predicted under future climate scenario SSP5-8.5 (n = 34). Currently, 54 species of Fagaceae have been recorded in a single grid cell (Fig. 1, Supplementary Table 2). Furthermore, we can infer that the species richness was high in Guangxi Province during the LGM, but it has gradually shifted to Yunnan Province during the present period. There was a gradual shift in the species richness towards the north and west, possibly at the higher elevations (Fig. 1a–f).

**Fig. 2:**
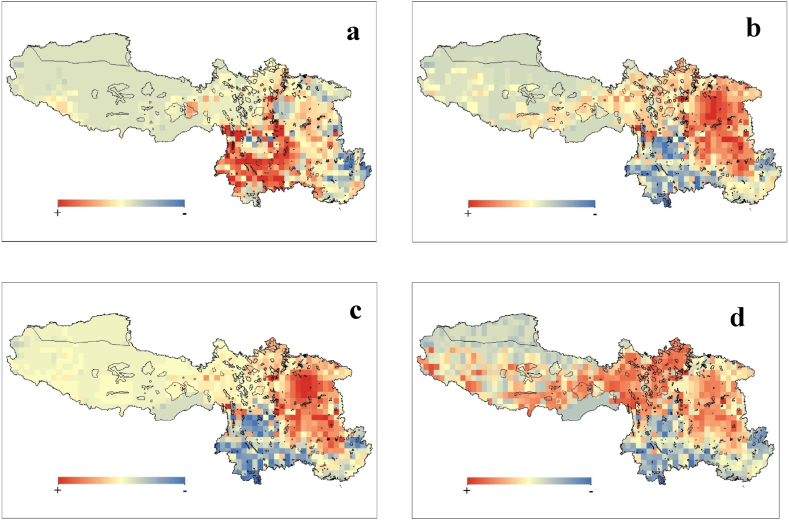
Change in species richness (ΔSR) between the present and future under climate change scenario- SSP1-2.6 (a), SSP2-4.5 (b), SSP3-7.0 (c), and SSP5-8.5 (d). The black boundary line represents the protected areas.

Similarly, pattern was noticed in the change in species richness (ΔSR) across time different climate scenarios (Fig. 2a–d). The findings show that during the worst climatic scenario (SSP5-8.5), the Tibetan Autonomous Regions will show positive change in species compared to other regions.

### 3.2 Spatial pattern of *β*-diversity and its components

**Fig. 3:**
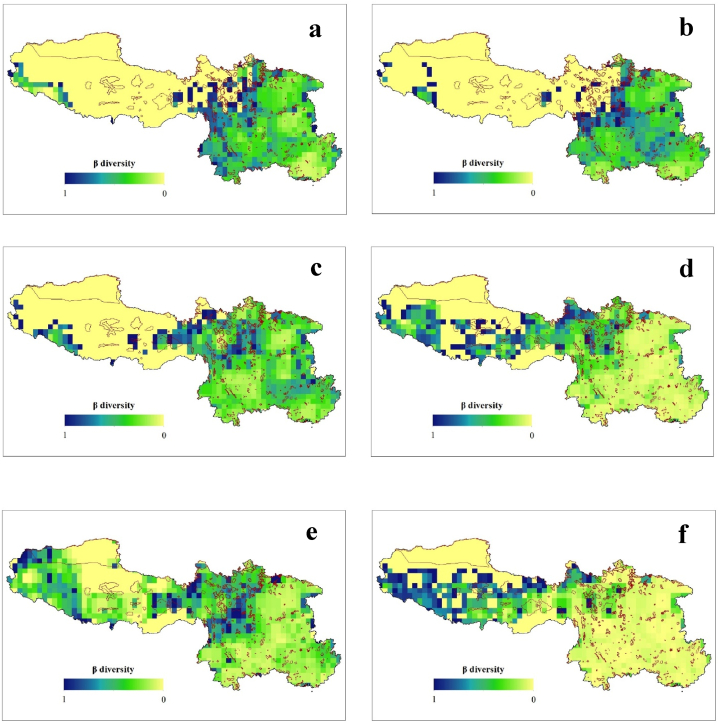
Geographic pattern of Sørensen-based pairwise dissimilarity (*β*_SOR_) of Fagaceae in Southwestern China during the historical (LGM) (a), present (b) and future (c–f) under climate change scenario- SSP1-2.6 (c), SSP2-4.5 (d), SSP3-7.0 (e), and SSP5-8.5 (f). The red boundary line represents the protected areas.

**Fig. 4:**
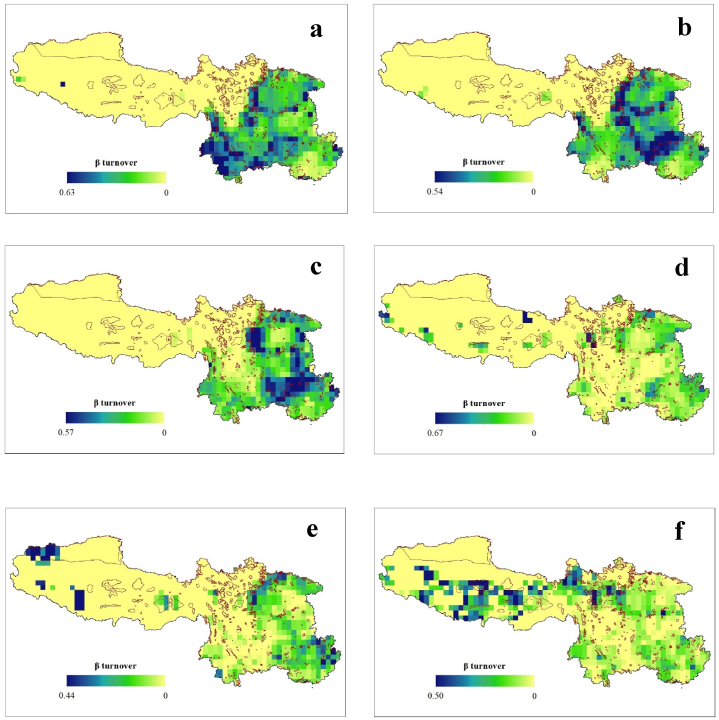
Historical (LGM) (a), Present (b) and Future (c–f) geographic pattern of true turnover (β_SIM_) of Fagaceae in Southwestern China. The red boundary line represents the protected areas.

**Fig. 5:**
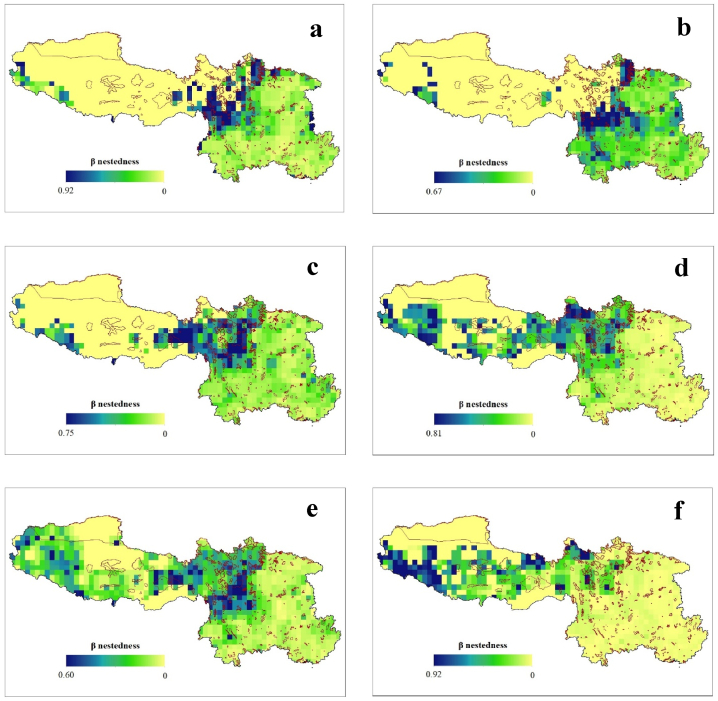
Historical (LGM) (a), Present (b) and Future (c–f) geographic pattern of nestedness (β_NES_) of Fagaceae in Southwestern China. The red boundary line represents the protected areas.

Among the neighboring grid cells in SW China, the geographic patterns of the total *β*-diversity (*β*_SOR_) and its components, i.e., the turnover (*β*_SIM_) and nestedness (*β*_NES_), exhibit a high level of congruency from the LGM to the future. The *β*_SOR_ values were higher during the LGM (mean ± SD = 0.368 ± 0.206), followed by the present period (0.358 ± 0.190), while the lowest values will occur in the future under scenario SSP3-7.0 (0.145 ± 0.115) (Fig. 3). Additionally, we observed weak correlations (Spearman's rank correlation: r_s_ ≤ ±0.75) between the different components of the *β*-diversity, except between *β*_SOR_ and *β*_NES_ under future climate change scenarios SSP2-4.5 and 3–7.0 scenarios. Overall, *β*_SOR_ exhibited higher values in Sichuan Province in all of the geological periods. Interestingly, in the future, we also predict that there will be elevated *β*_SOR_ values of Fagaceae in the Tibetan region. Similar patterns were observed for *β*_SIM_ and *β*_NES_ (Fig. 4, Fig. 5).

**Fig. 6:**
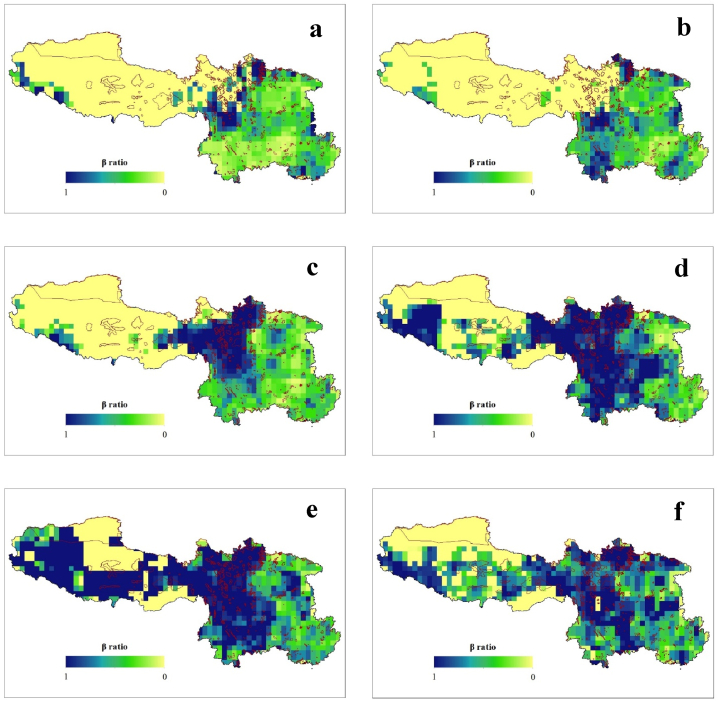
Geographic pattern of beta-ratio (*β*_ratio_) of Fagaceae in Southwestern China during the historical (LGM) (a), present (b) and future (c–f) under climate change scenario- SSP1-2.6 (c), SSP2-4.5 (d), SSP3-7.0 (e), and SSP5-8.5 (f). The red boundary line represents the protected areas.

Furthermore, the *β*_ratio_, which quantifies the contribution of *β*_NES_ to the overall *β*-diversity (*β*_SOR_), exhibited variations. We found that the mean *β*_ratio_ was less than 0.5 during the LGM (0.362 ± 0.246) and present (0.439 ± 0.235), but it will be greater than 0.5 under the different future climate change scenarios, such as SSP1-2.6 (0.538 ± 0.30), SSP2-4.5 (0.723 ± 0.293), SSP3-7.0 (0.778 ± 0.281) and SSP5-8.5 (0.615 ± 0.251) (Fig. 6c–f). During the LGM, most of the grid cells in the Sichuan Province had values of greater than 0.5 compared to the other regions. Remarkably, at present, the higher *β*_ratio_ values occur in the southern part of Sichuan and Yunnan Province. In contrast, as shown in Fig. 6, there will be a gradual increase in the *β*_ratio_ from east to west in SW China in the future. Additionally, in addition to Sichuan and Yunnan, the Tibetan region will also have higher *β*_ratio_ values, while the Guangxi, Guizhou and Chongqing regions will have *β*_ratio_ values of less than 0.5.

### 3.3 Drivers of *α-* and *β-*diversity during the LGM, the present and the future

As shown in Table 1, BIO 15, BIO 18, BIO 19, the mean elevation (Elv), and the elevation range (ER) were found to be significant in determining the species richness pattern (*α*-diversity). These variables collectively accounted for 77 % of the variance in the richness pattern of Fagaceae in SW China. For the *β*–diversity (*β*_SOR_) analysis, the best-fitted model included BIO 19, the mean elevation, and the elevation range as significant variables, explaining 34 % of the variance. Similarly, all of the climatic variables significantly contributed to explaining 39 % of the variance in the species turnover (*β*_SIM_). In contrast, during the LGM, the predictors BIO 18, and 19 and the elevation range were identified as key factors in explaining 39 % and 23 % variance in nestedness (*β*_NES_) and beta ratio (*β*_ratio_) respectively.

**Table 1:** Best-fitted SARerr models predicting, historical (LGM) species richness (α-diversity), Sørensen-based pairwise dissimilarity (β_SOR_), and its underlying true turnover (β_SIM_), nestedness (β_NES_), and ratio (β_ratio_).

Variables	α*-diversity*	*β*_SOR_	*β*_SIM_	*β*_NES_	*β*_ratio_
**Climate**					
BIO 04	0.0005 ^NS^	–	**−0.0174**	**-**	**-**
BIO 15	**−2.1443**	0.0019 ^NS^	**−0.0109**	–	–
BIO 18	**10.5291**	**-**	**0.0566**	**−0.0185**	**−0.0534**
BIO 19	**5.3925**	**−0.1032**	**−0.0076**	**−0.0685**	**−0.0190**
**Topographic**					
Elv	**−1.8161**	**−0.010**	**−**0.003 ^NS^	**−**0.008 ^NS^	–
ER	**−1.1047**	**0.059**	0.008 ^NS^	**0.024**	**0.0136**

**minRSA**	1.01	0.98	1.48	1.25	1.48
***Pseudo-R***^***2***^	0.77	0.34	0.39	0.39	0.23

For abbreviations of predictor variables please refer to Supplementary Table 1.1. The number in the bold indicate the significant values (p < 0.05), while ^NS^ denotes no significance.

The current species richness of Fagaceae is significantly influenced by four climatic variables (BIO 03, 07, 18, 19) and seed volume as presented in Table 2. These identified variables collectively account for 53 % of the variance in the current richness pattern. In terms of *β*_SOR_, the variables BIO 15, 18, 19 and leaf length explained 30 % of the variance. Furthermore, climatic factors and plant traits contribute to explaining 30 %, 30 %, and 16 % of the variance in *β*_SIM_. *β*_NES_, and *β*_ratio_, respectively.

**Table 2:** Best-fitted SARerr models predicting, current species richness (*α*-diversity), Sørensen-based pairwise dissimilarity (*β*_SOR_), and its underlying true turnover (*β*_SIM_), nestedness (*β*_NES_) and ratio (*β*_ratio_).

Variables	α*-diversity*	*β*_SOR_	*β*_SIM_	*β*_NES_	*β*_ratio_
**Climatic**					
BIO 03	**−0.4747**	**-**	**−0.0628**	**0.0302**	**0.0911**
BIO 07	**−4.5289**	–	**−0.0425**	–	–
BIO 15	–	**0.0521**	–	–	**−0.0362**
BIO 18	**2.5303**	**−0.0476**	**0.0217**	−0.0051 ^NS^	**0.0065**
BIO 19	**1.5685**	**−0.0262**	**−0.0291**	**−0.0333**	**−0.0341**
**Topographic**					
Elv	**−**0.1655 ^NS^	–	–	0.0076 ^NS^	–
ER	0.0549 ^NS^	–	–	–	–
GD	0.7316 ^NS^	–	0.0089 ^NS^	0.0035 ^NS^	–
**Plant traits**					
Tree height	–	–	0.0053^NS^	–	–
Leaf length	0.5935 ^NS^	**−0.0199**	–	**−0.0274**	–
Seed volume	**−0.8754**	−0.0126 ^NS^	**−0.0121**	**−0.0101**	**−0.0347**

**minRSA**	1.01	1.17	1.59	1.13	1.38
***Pseudo-R***^***2***^	0.53	0.30	0.30	0.30	0.16

For abbreviations of predictor variables please refer to Supplementary Table 1.1. The number in the bold indicate the significant values (p < 0.05), while ^NS^ denotes no significance.

In the SSP1-2.6 climate change scenario, BIO 04 and BIO 18 emerged as the most influential predictors of species richness, collectively explaining 58 % of the variance. Moreover, under all of the climate change scenarios (as shown in Table 3), the climatic variables are significant predictors, contributing to the determination of the *β-*diversity components and explaining 18 %–60 % of the variances. Similar patterns occur under the other climate change scenarios (Table 4, Table 5, Table 6). For instance, the variations in the species richness patterns are 80 %, 91 %, and 70 % for SSP2-4.5, SSP3-7.0, and SSP5-8.5, respectively. Notably, the highest variance in *β*_SOR_ occurs under scenario SSP5-8.5, reaching 50 %.

**Table 3:** Best fitted SARerr models predicting, future (*SSP1-2.6*) species richness (*α*-diversity), Sørensen-based pairwise dissimilarity (*β*_SOR_), and its underlying true turnover (*β*_SIM_), nestedness (*β*_NES_) and ratio (*β*_ratio_).

Variables	α*-diversity*	*β*_SOR_	*β*_SIM_	*β*_NES_	*β*_ratio_
**Climate**					
BIO 04	**−4.9409**	**−0.0331**	**0.0414**	**−0.0327**	**−0.1251**
BIO 18	**5.5818**	**−0.0120**	**0.5922**	**−0.0713**	**−0.1523**
**Topographic**					
Elv	**-**	**-**	**-**	**-**	**-**
ER	**-**	**-**	**-**	0.0077^NS^	0.0188^NS^

**minRSA**	2.57	1.22	1.75	2.89	3.54
***Pseudo-R***^***2***^	0.58	0.18	0.30	0.20	0.21

For abbreviations of predictor variables please refer to Supplementary Table 1.1. The number in the bold indicate the significant values (p < 0.05), while ^NS^ denotes no significance.

**Table 4:** Best fitted SARerr models predicting, future (*SSP2-4.5*) species richness (*α*-diversity), Sørensen-based pairwise dissimilarity (*β*_SOR_), and its underlying true turnover (*β*_SIM_), nestedness (*β*_NES_) and ratio (*β*_ratio_).

Variables	α*-diversity*	*β*_SOR_	*β*_SIM_	*β*_NES_	*β*_ratio_
**Climate**					
BIO 03	**−4.3342**	**0.0252**	**−0.007**	**0.0560**	**0.1576**
BIO 07	**−8.2391**	**0.0631**	**-**	**0.0596**	**-**
BIO 15	**−2.7746**	**0.0675**	**-**	**-**	**−0.1509**
BIO 19	**-**	**0.0227**	**0.002**	**-**	**−0.1630**
**Topographic**					
Elv	**-**	**-**	**-**	**-**	**-**
ER	**-**	**-**	**-**	**-**	**-**

**minRSA**	1.37	0.71	0.40	1.16	1.11
***Pseudo-R***^***2***^	0.80	0.49	0.03	0.43	0.38

For abbreviations of predictor variables please refer to Supplementary Table 1.1. The number in the bold indicate the significant values (p < 0.05), while ^NS^ denotes no significance.

**Table 5:** Best fitted SARerr models predicting, future (*SSP3-7.0*) species richness (*α*-diversity), Sørensen-based pairwise dissimilarity (*β*_SOR_), and its underlying true turnover (*β*_SIM_), nestedness (*β*_NES_) and ratio (*β*_ratio_).

Variables	α*-diversity*	*β*_SOR_	*β*_SIM_	*β*_NES_	*β*_ratio_
**Climate**					
BIO 03	**−4.4235**	**0.0509**	**−0.0073**	**0.0606**	**0.2055**
BIO 04	**−5.5551**	**0.0433**	**-**	**0.0410**	**0.0521**
BIO 15	**−4.7167**	**−0.0342**	−0.0002^NS^	**−0.0467**	**−0.1811**
BIO 18	**0.8986**	**0.0243**	**0.0059**	**0.0166**	**-**
BIO 19	**0.7717**	**-**	**0.0036**	**−0.0180**	**−0.0601**
**Topographic**					
Elv	**-**	**-**	**-**	**-**	**-**
ER	**−**0.1873 ^NS^	**-**	**-**	**-**	**-**

**minRSA**	1.30	1.42	0.56	1.22	1.01
***Pseudo-R***^***2***^	0.91	0.24	0.13	0.35	0.25

For abbreviations of predictor variables please refer to Supplementary Table 1.1. The number in the bold indicate the significant values (p < 0.05), while ^NS^ denotes no significance.

**Table 6:** Best fitted SARerr models predicting, future (*SSP5-8*.*5*) species richness (*α*-diversity), Sørensen-based pairwise dissimilarity (*β*_SOR_), and its underlying true turnover (*β*_SIM_), nestedness (*β*_NES_) and ratio (*β*_ratio_).

Variables	α*-diversity*	*β*_SOR_	*β*_SIM_	*β*_NES_	*β*_ratio_
**Climate**					
BIO 03	**−0.6817**	**0.0499**	**0.0236**	**0.0405**	**0.0428**
BIO 18	**1.8834**	**−0.1874**	**−0.0735**	**−0.0798**	**0.0665**
BIO 19	**2.1814**	**-**	**-**	**-**	**-**
**Topographic**					
Elv	**-**	0.0099 ^NS^	–	**-**	**-**
ER	**-**	**-**	**-**	**-**	**-**

**minRSA**	0.71	0.96	0.79	0.81	0.53
***Pseudo-R***^***2***^	0.70	0.597	0.18	0.24	0.15

For abbreviations of predictor variables please refer to Supplementary Table 1.1. The number in the bold indicate the significant values (p < 0.05), while ^NS^ denotes no significance.

## 4 Discussion

This paper presents the first comprehensive analysis of the *α*- (species richness) and *β*-diversity of Fagaceae during the historical (LGM) and present, as well as future projections under various SSPs, across the southwestern region of China. The analysis was performed at a spatial resolution of 50 × 50 km^2^, encompassing a wide geographical area to ensure representative results. Our objective was to comprehend the spatial distribution and the patterns of species diversity of this ecologically significant plant family. We examined diversity patterns across historical, current and future time periods to gain a more comprehensive perspective. Furthermore, in this study, we successfully identified significant potential predictors at the regional scale, providing insights into how the diversity of Fagaceae has change over time and how it may respond to potential changes in climate, topography, and trait conditions. The three key findings of our study are as follows. First, there is a decline in the species richness (*α*-diversity) from the LGM to the future. Additionally, there is a gradual shift in the highest species richness from Guangxi during the LGM to Yunnan at present, and further north and west in the future. Moreover, the *β*-diversity was high in Sichuan Province during the LGM and remains high at present, but it is projected to shift westward toward the Tibetan region in the future. Second, climatic variables are more prominent in explaining the *α* and *β*-diversity of Fagaceae across SW China. Third, our findings support the idea that the species turnover played an important role in driving the differences in the species composition among sites during the LGM and present periods, while nestedness will be a dominant factor in the future.

### 4.1 Change in *α*-diversity and its causes

The species composition within a geographic area is the outcome of various historical and ecological processes, both within local communities and across different regions [19,22,24]. Although we observed consistency in the number of species during the LGM (n = 120), present period, and under climate change scenario SSP1-2.6, there are some notable differences in the distribution from the historical period to the present period and into the future (Fig. 1, Fig. 2, Fig. 3, Fig. 4, Fig. 5). Similar to the results presented by Liang et al. [25] for plants, we also predict a gradual decrease in the species richness in the future, which will be accompanied by an expansion of the species' distribution areas. These shifts in the species richness and distribution patterns are unlikely to be solely controlled to climatic and topographic variables. Similar findings have been reported by Liao et al. [15] and Dakhil et al. [24] for Lauraceae and conifers distributions, respectively. During the LGM, the precipitation in the warmest quarter (BIO18) and the precipitation in the coldest quarter (BIO19) had positive effects on the species richness. In contrast, factors such as precipitation seasonality (BIO15), mean elevation, and elevation range had negative influences on determining the richness pattern. Moreover, in the present period, both the climatic and plant trait (seed volume) have significant effect. On the other hand, the climatic variables are projected to have significant effect in the future. SW China, which is considered the refugium for plants [51], was characterized by increased precipitation during the warmest and coldest quarters of the year during the LGM, thus, favoring the Fagaceae species in lower elevation regions of Yunnan and Guangxi. This result is also supported by palaeodistribution model [52], where we found that Yunnan is rich in potential distribution of Fagaceae in the past (Supplementary Fig. 1.3). As expected, the species were confined to lower elevations during this period, but they are anticipated to shift toward higher elevations in the future, expanding westwards toward the Qinghai-Tibetan Regions (refer to Fig. 1). Similarly, the climatic variables in the Yunnan and Sichuan region of SW China might also favor a high species richness. As climatic variables have been identified as the primary drivers of the current distribution of gymnosperms in China [19], these areas serve as refugia during climatic fluctuation and glaciations.

Furthermore, the warming climate in the future is anticipated to transform alpine habitats into emerging biodiversity hotspots for species that currently inhabit lower elevation area [53]. One reason for the decrease in species richness is linked to biological invasion, which leads to an increase in nonnative species. While the introduction of nonnative plants can potentially increase the *α*-diversity, the invasion of such plants frequently leads to a reduction in the diversity of the indigenous species, particularly within limited geographical areas. Rapid climate change is currently inducing significant alterations in the eco-climatic conditions, posing a global threat to species diversity [10]. Therefore, identifying the factors that impact biodiversity within a region is significant for projecting how ecosystems will react to shifts in the environment. This potential shift highlights the importance of prioritizing the aspect of the conservation planning of biodiversity in the SW China. Expanding protected areas, implementing flexible management practices, and establishing monitoring programs to track ecosystem responses to environmental shifts may help ensure the conservation of plant species.

### 4.2 Relative role of *β*-diversity and its components

Numerous studies have investigated the effects of climatic, topographic, and edaphic factors that determine the compositional dissimilarity between communities [[2], [3], [4],^15^,^17^,^18^], but no study has reported the importance of these factors in SW China during three different time period, even though the ecosystem in this region changes in different dimension. It is notable that the highest *β*_SOR_ occurred in the Sichuan region during the LGM and the present, while in the future, under the SSP1-2.6 scenario, the *β*_SOR_ will be highest in the Tibetan region. Complex interactions between the *β*-diversity and predictors lead to changes in the community composition from one location to another. Interestingly, the *β*-diversity during the LGM was controlled by the positive effect of the elevation range and the negative effects of precipitation in the coldest quarter (BIO19) and the mean elevation. Thus, elevation and climatic gradients create heterogeneity in habitats that support the adaptation of a distinct community. In the future, the climatic variables will have a pronounced effect on the *β*-diversity. Climatic conditions play a critical role in shaping ecological communities by acting as selective filters that determine which species can successfully establish themselves and persist within a given habitat [17,20]. A high *β-*diversity can indicate areas of high ecological importance, as they may harbor unique or rare species that have specialized adaptations to specific local conditions. Conservation efforts may need to consider preserving these areas to maintain overall biodiversity particularly for mountain communities.

Our findings reveal a novel linkage between the climatic gradient and the component of the *β*-diversity for Fagaceae species. Spatial turnover and nestedness are recognized as two antithetical processes leading to community composition variance [7]. However, these components of *β*-diversity are not expected to be evenly distributed across geographic space (Fig. 3, Fig. 4, Fig. 5). The variations in the turnover and nestedness across SW China tend to be opposite that of *β*-diversity. Similar patterns have been reported for angiosperms [4,54] in China. Additionally, the climatic variables were significant in determining the diversity of Fagaceae in all three studied time periods (Table 1, Table 2, Table 3, Table 4, Table 5, Table 6). A possible explanation for expansion of the *β*-diversity toward the Tibetan Plateau is climate change. The Fagaceae family includes species found in the transition zone between the tropical and temperate regions in China [29]. Future climatic changes will create suitable habitats for these species, even at higher elevations, enabling their adaptation to these regions without the constraints of their dispersal capacities. This is common among animals [2,8,23], and has been reported in numerous previous studies in plants [11,15,22,24,25]. This correlation is also strongly reflected in our own findings. The variations in the dispersal patterns among species, whereby certain species are unable to reach all appropriate habitats, can give rise to *β*-diversity [55]. In a scenario with boundless dispersal and comparable abiotic conditions, every species would exist across all locations, resulting in nearly zero *β*-diversity [56]. Therefore, according to the community assembly history theory [57], when there is a disparity in the chronological sequence of species arrivals, it can lead to the emergence of multiple stable equilibria in homogeneous environments. This, in turn, contributes to increased *β*-diversity [55].

Moreover, the geographic distance has a significant positive relationship with *β*_SIM_ during the present period (Table 2). The geographic distance is often used as a proxy for dispersal in ecological and biogeographical studies [58]. The distance-decay relationship [59] describes how the similarity of the species compositions of two sites decreases as the geographic distance between them increases. This relationship is often observed in plant communities and reflects the influence of dispersal limitation [56,59]. When species have limited dispersal abilities, they are more likely to be present in nearby locations and are less likely to reach sites that are far apart. As a result, the similarity in the species compositions decreases with increasing geographic distance, leading to a higher *β*-diversity [15]. In terms of dispersal, some Fagaceae species produce seeds encased in acorns, nuts, or burrs that are well suited for various modes of dissemination [29]. Our findings suggested that seed volume has negative impact on the component of *β*-diversity (Table 2). Study showed that post-dispersal seed predation is a particularly strong consumer interaction that can shape plant population dynamics, spatial distribution, and community structure [60]. Animals such as squirrels, birds, and even larger mammals play a crucial role in transporting these seeds over various distances, which contributes to the species' ability to colonize new areas and maintain genetic diversity. Fagaceae seed dispersal, with its influence on introducing new species to different habitats, is consistent with the observation that habitat conditions may vary over geographic distances. Consequently, the spatial limitation of seed dispersal may contribute to a pronounced turnover of the species composition, as habitats more distant from seed sources experience higher levels of species change.

### 4.3 *β*_ratio_ and its implication for conservation

Our result suggest that a prevalence of species turnover contributed to most of the *β*-diversity during the LGM and in the present period (with a *β*_ratio_ of less than 0.5), whereas in the future, nestedness will likely be the predominant component (with a *β*_ratio_ of greater than 0.5). The nestedness concept within species assemblages implies that species exclusion is influenced by environmental filtering, while the turnover signifies species replacement owing to environmental factors or spatial constraints [5,22,23]. One potential explanation for the dominance of nestedness in explaining the *β*-diversity of Fagaceae in the future could be linked to the influential role of selective extinction. Selective extinction, caused by environmental filtering such as climate change, plays a significant role in shaping the plant distribution. It acts as a barrier for some plants while allowing others to thrive in high abiotic stress environments. In extreme scenarios, a site with fewer species could exhibit a unique assemblage compared to a more species-rich location, underscoring the importance of conserving the species-poor site to enhance overall diversity. Conversely, if the species-poor site shares species with the species-rich area, focusing on conserving the latter becomes crucial [13,23].

Moreover, the *β*_ratio_ plays a significant role in conservation [22]. A *β*_ratio_ of greater than 0.5 indicates a relatively high degree of nestedness in the species composition between different habitats or geographical areas [5,13,22]. Nestedness patterns emerge when the gain or loss of species causes species-poor sites to resemble a strict subset of the species found in species-rich sites. Therefore, in the future, as the *β-*diversity mainly consists of the nestedness component, a single large nature reserve should be suitable for conservation. Conversely, when the *β*_ratio_ is less than 0.5, there is a greater turnover of species between sites [13,22]. Turnover takes place when new sites experience a substitution of existing species with different ones. If the turnover component dominates the *β*-diversity, it is more effective to establish several small nature reserves [10,13]. Thus, at present, in the conservation of Fagaceae in SW China, we must target smaller multiple sites.

## 5 Conclusion

Our comprehensive investigation of the dynamics of the *α-* (species richness) and *β*-diversity of Fagaceae during the LGM, present, and future periods in SW China has yielded valuable insights into the spatial distribution and patterns of these ecologically significant plants. Our results provide a deeper understanding of not only the present biodiversity but also of the historical and future biodiversities. Notably, the species richness declined from the LGM to the present period and will continue to decline in the future, underlining the historical and future viewpoints in biodiversity analyses. Furthermore, our findings indicated that climatic variables play a dominant role in explaining the variability of the *α-* and *β*-diversity. Interestingly, we found that, while species turnover primarily shaped the *β*-diversity during the LGM and the present, under future scenarios, the nestedness component will be more important. Our results have implications for conservation efforts in SW China. They highlight the need to consider historical, current, and future dynamics when developing conservation strategies for Fagaceae and other plant families. As climate change continues to shape ecosystems, understanding the interplay between species richness, *β*-diversity components, and ecological processes are becoming increasingly vital for effective biodiversity management. By identifying the drivers of diversity changes and recognizing the roles of turnover and nestedness, the findings of our study provide a guidelines and decision-support tools for relevant decision makers and experts, to implement efficient conservation and management programs in the global hotspot in SW China.

## CRediT authorship contribution statement

**Bikram Pandey:** Writing – review & editing, Writing – original draft, Visualization, Validation, Software, Methodology, Formal analysis, Data curation, Conceptualization. **Fengying Zhang:** Writing – review & editing, Data curation. **Basu Dev Poudel:** Writing – original draft, Visualization, Formal analysis. **Rong Li:** Writing – review & editing, Investigation. **Mohammed A. Dakhil:** Writing – review & editing, Writing – original draft. **Bishal Gurung:** Writing – review & editing, Writing – original draft, Visualization. **Zhaxi Luobu:** Writing – review & editing. **Yawen Gan:** Writing – review & editing. **Ziyan Liao:** Writing – original draft, Funding acquisition, Formal analysis. **Lin Zhang:** Supervision, Project administration, Investigation, Funding acquisition, Conceptualization.

## Data availability statement

The data that support the findings of this study are openly available in Dryad and can be download from https://doi.org/10.5061/dryad.2rbnzs7vq.

## Funding

This work was supported by the 10.13039/501100001809National Natural Science Foundation of China (grant number 32201424, 2023–2025) and West Light Foundation of Chinese Academy of Sciences (CAS) (2022XBZG_XBONXZ_A_003).

## Declaration of competing interest

The authors declare that they have no known competing financial interests or personal relationships that could have appeared to influence the work reported in this paper.
