# Supplementary material for: From α-to β-diversity: Understanding the historical, present, and future diversity patterns of Fagaceae in Southwestern China
Source: Heliyon. 2024 Dec 31;11(2):e41474. doi: 10.1016/j.heliyon.2024.e41474 (PMC11783019; doi:10.1016/j.heliyon.2024.e41474)
Supplement: Multimedia component 1 [file mmc1.docx]

**Supplementary Material 1**


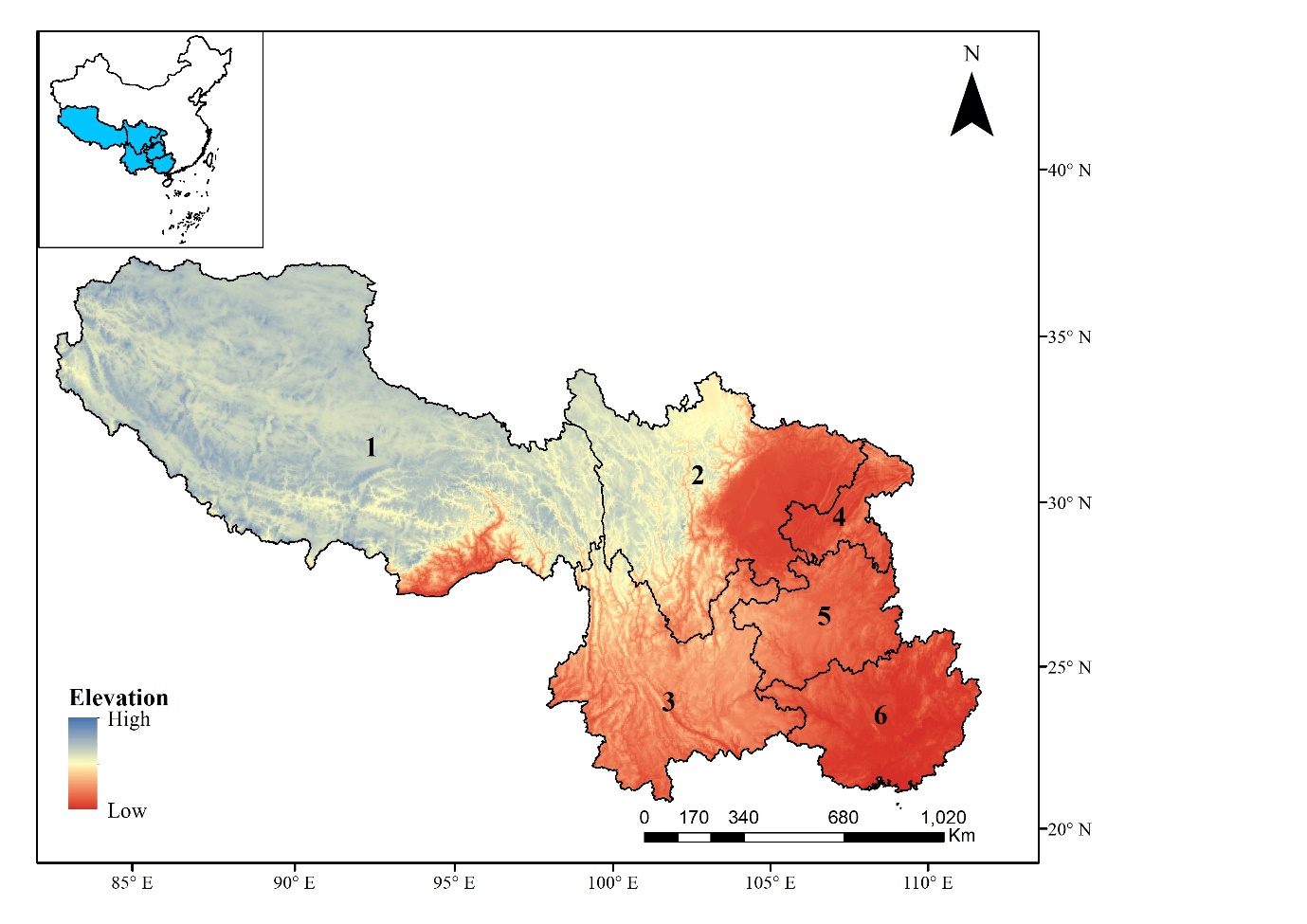


Figure 1.1: The map of South-western China showing – 1. Tibet Autonomous region, 2. Sichuan Province, 3. Yunnan Province, 4. Chongqing Municipality, 5. Guizhou Province, 6. Guangxi Autonomous Region


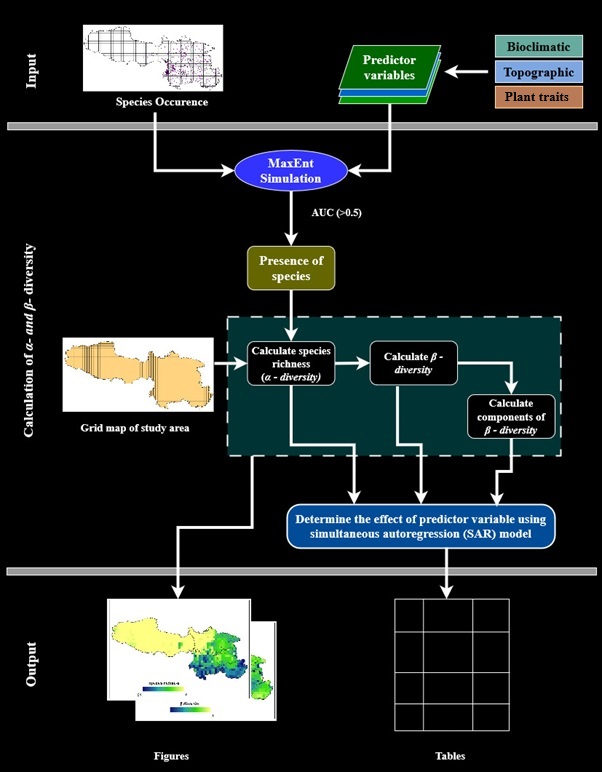


Figure 1.2: Flowchart showing statistical and spatial modelling analyses


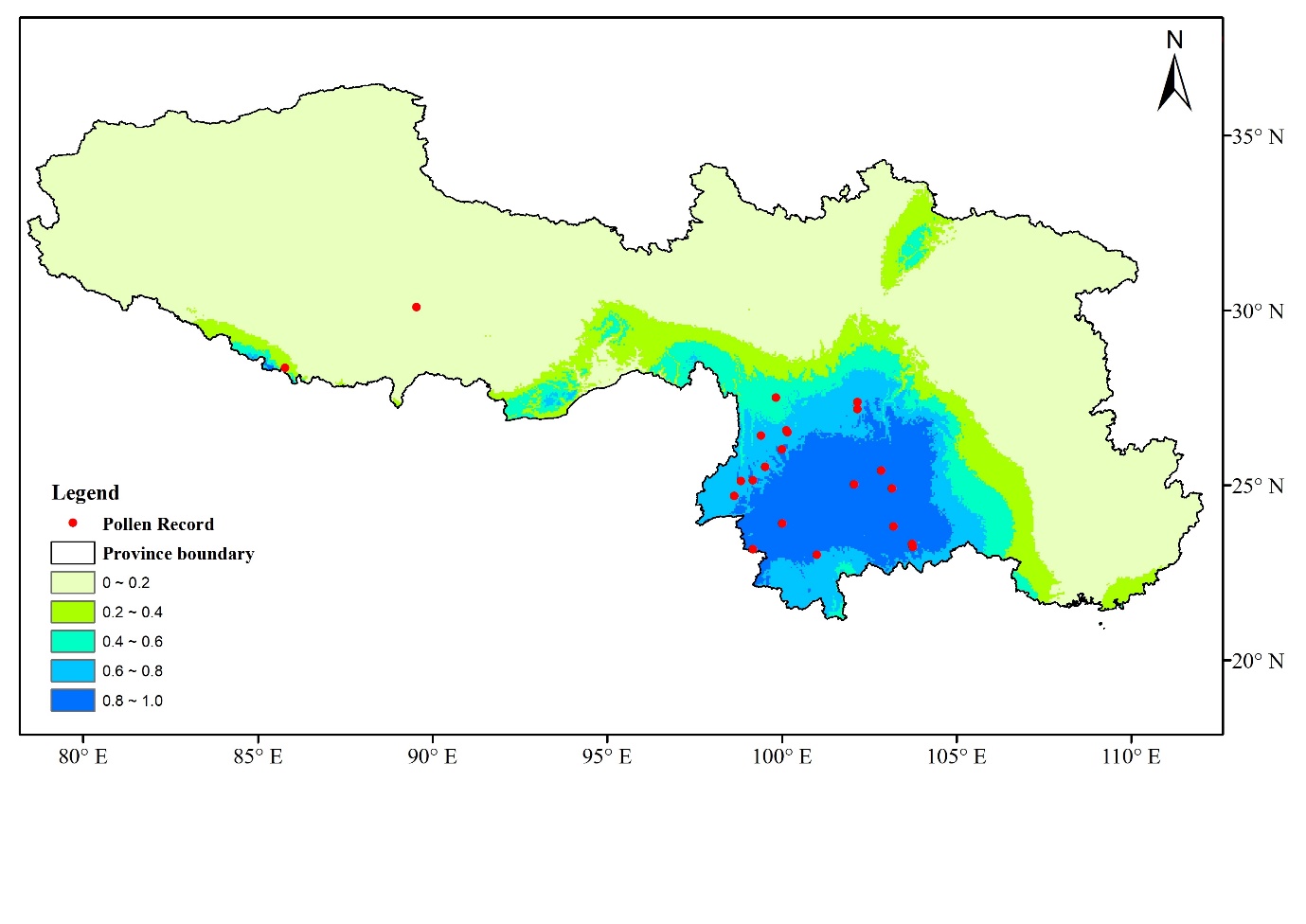


Figure 1.3: Potential distribution of Fagaceae in the past supported by pollen records. The data source of pollen record is cited in the main text.

Table 1.1: List of predictor variables, their abbreviation and units, and the data sources used in the research.

|  | | **Variables** | **Unit** | **Source** |
| --- | --- | --- | --- | --- |
| **Climate** | | | | |
|  | BIO 01 | Annual Mean Temperature | °C | CHELSA, WorldClim |
|  | BIO 02 | Mean Diurnal Range (Mean of monthly (max temp - min temp)) | °C | CHELSA, WorldClim |
|  | BIO 03 | Isothermality (BIO2/BIO7) (×100) | % | CHELSA, WorldClim |
|  | BIO 04 | Temperature Seasonality (standard deviation ×100) | °C | CHELSA, WorldClim |
|  | BIO 05 | Max Temperature in Warmest Month | °C | CHELSA, WorldClim |
|  | BIO 06 | Min Temperature in Coldest Month | °C | CHELSA, WorldClim |
|  | BIO 07 | Annual Temperature Range (BIO5-BIO6) | °C | CHELSA, WorldClim |
|  | BIO 08 | Mean Temperature in Wettest Quarter | °C | CHELSA, WorldClim |
|  | BIO 09 | Mean Temperature in Driest Quarter | °C | CHELSA, WorldClim |
|  | BIO 10 | Mean Temperature in Warmest Quarter | °C | CHELSA, WorldClim |
|  | BIO 11 | Mean Temperature in Coldest Quarter | °C | CHELSA, WorldClim |
|  | BIO 12 | Annual Precipitation | mm | CHELSA, WorldClim |
|  | BIO 13 | Precipitation in Wettest Month | mm | CHELSA, WorldClim |
|  | BIO 14 | Precipitation in Driest Month | mm | CHELSA, WorldClim |
|  | BIO 15 | Precipitation Seasonality (Coefficient of variation) | % | CHELSA, WorldClim |
|  | BIO 16 | Precipitation in Wettest Quarter | mm | CHELSA, WorldClim |
|  | BIO 17 | Precipitation in Driest Quarter | mm | CHELSA, WorldClim |
|  | BIO 18 | Precipitation in Warmest Quarter | mm | CHELSA, WorldClim |
|  | BIO 19 | Precipitation in Coldest Quarter | mm | CHELSA, WorldClim |
| **Topographic** | | |  |  |
|  | Elv | Mean Elevation | m | CHELSA, SRTM |
|  | ER | Elevation Range | m | CHELSA, SRTM |
|  | GD | Geographical Distance | km | CHELSA, SRTM |
| **Plant traits** | | |  |  |
|  | Tree height | Tree height | m | [27] [29] |
|  | Leaf length | Leaf length | cm | [27] [29] |
|  | Leaf width | Leaf width | cm | [27] [29] |
|  | Seed volume | Seed volume | cm^3^ | [27] [28] [29] |

All the data sources are mentioned in the main text.

Table 1.2: List of variables used for analysis after the removal of highly correlated variables (r > 0.8) using Pearson correlation tests between the predictor variables.

|  | | Climatic | Topographic | Plant traits |
| --- | --- | --- | --- | --- |
| Historical | | BIO 04, 15, 18, 19 | Elv, ER |  |
| Current | | BIO 03, 07, 15, 18, 19 | Elv, ER, GD | Tree height, leaf length, seed volume |
| Future | SSP 1.26 | BIO 04, 18 | Elv, ER |  |
|  | SSP 2.45 | BIO 03, 07, 15, 19 | Elv, ER |  |
|  | SSP 3.70 | BIO 03, 04, 15, 18, 19 | Elv, ER |  |
|  | SSP 5.85 | BIO 03, 18, 19 | Elv, ER |  |

Please refer to Supplementary Table 1 for details about the predictors.

Table 1.3: Descriptive information on biodiversity pattern in all studied time period.

|  | | Total number of species | Total number of grid cells in which species occurs | Highest number of species in a grid | Number of species lost |
| --- | --- | --- | --- | --- | --- |
| Historical | | 120 | 599 | 64 | 0 |
| Current | | 120 | 538 | 54 | 0 |
| Future | SSP 1.26 | 120 | 681 | 55 | 0 |
|  | SSP 2.45 | 54 | 838 | 40 | 66 |
|  | SSP 3.70 | 57 | 1043 | 41 | 63 |
|  | SSP 5.85 | 49 | 1023 | 34 | 71 |
