# Supplementary material for: From α-to β-diversity: Understanding the historical, present, and future diversity patterns of Fagaceae in Southwestern China
Source: Heliyon. 2024 Dec 31;11(2):e41474. doi: 10.1016/j.heliyon.2024.e41474 (PMC11783019; doi:10.1016/j.heliyon.2024.e41474)
Supplement: Multimedia component 2 [file mmc2.docx]

**SUPPLEMENT MATERIAL 2**

**Table 2.1: Correlation among predictor variables from Past (Last Glacial Maximum; LGM).**

|  | **Bio01** | **Bio02** | **Bio03** | **Bio04** | **Bio05** | **Bio06** | **Bio07** | **Bio08** | **Bio09** | **Bio10** | **Bio11** | **Bio12** | **Bio13** | **Bio14** | **Bio15** | **Bio16** | **Bio17** | **Bio18** | **Bio19** | **Er** | **Elv** | **Gd** |
| --- | --- | --- | --- | --- | --- | --- | --- | --- | --- | --- | --- | --- | --- | --- | --- | --- | --- | --- | --- | --- | --- | --- |
| **Bio01** | 1 |  |  |  |  |  |  |  |  |  |  |  |  |  |  |  |  |  |  |  |  |  |
| **Bio02** | -0.751 | 1 |  |  |  |  |  |  |  |  |  |  |  |  |  |  |  |  |  |  |  |  |
| **Bio03** | -0.270 | 0.778 | 1 |  |  |  |  |  |  |  |  |  |  |  |  |  |  |  |  |  |  |  |
| **Bio04** | -0.732 | -0.723 | -0.782 | 1 |  |  |  |  |  |  |  |  |  |  |  |  |  |  |  |  |  |  |
| **Bio05** | 0.950 | -0.833 | -0.513 | -0.023 | 1 |  |  |  |  |  |  |  |  |  |  |  |  |  |  |  |  |  |
| **Bio06** | 0.995 | -0.769 | -0.261 | -0.348 | 0.932 | 1 |  |  |  |  |  |  |  |  |  |  |  |  |  |  |  |  |
| **Bio07** | -0.768 | 0.432 | -0.224 | 0.768 | -0.540 | -0.809 | 1 |  |  |  |  |  |  |  |  |  |  |  |  |  |  |  |
| **Bio08** | 0.961 | -0.825 | -0.426 | -0.149 | 0.959 | 0.953 | -0.656 | 1 |  |  |  |  |  |  |  |  |  |  |  |  |  |  |
| **Bio09** | 0.908 | -0.543 | -0.052 | -0.440 | 0.824 | 0.907 | -0.767 | 0.817 | 1 |  |  |  |  |  |  |  |  |  |  |  |  |  |
| **Bio10** | 0.969 | -0.852 | -0.489 | -0.078 | 0.994 | 0.957 | -0.610 | 0.974 | 0.840 | 1 |  |  |  |  |  |  |  |  |  |  |  |  |
| **Bio11** | 0.983 | -0.646 | -0.096 | -0.490 | 0.879 | 0.983 | -0.856 | 0.913 | 0.923 | 0.907 | 1 |  |  |  |  |  |  |  |  |  |  |  |
| **Bio12** | 0.878 | -0.745 | -0.347 | -0.208 | 0.839 | 0.872 | -0.664 | 0.846 | 0.777 | 0.866 | 0.847 | 1 |  |  |  |  |  |  |  |  |  |  |
| **Bio13** | 0.839 | -0.653 | -0.227 | -0.301 | 0.767 | 0.835 | -0.693 | 0.805 | 0.764 | 0.801 | 0.829 | 0.946 | 1 |  |  |  |  |  |  |  |  |  |
| **Bio14** | 0.688 | -0.678 | -0.481 | 0.087 | 0.727 | 0.671 | -0.378 | 0.671 | 0.637 | 0.740 | 0.617 | 0.826 | 0.685 | 1 |  |  |  |  |  |  |  |  |
| **Bio15** | -0.411 | 0.505 | 0.402 | -0.128 | -0.465 | -0.415 | 0.207 | -0.356 | -0.362 | -0.465 | -0.355 | -0.467 | -0.216 | -0.627 | 1 |  |  |  |  |  |  |  |
| **Bio16** | 0.864 | -0.655 | -0.203 | -0.345 | 0.780 | 0.861 | -0.734 | 0.824 | 0.787 | 0.815 | 0.860 | 0.959 | 0.990 | 0.692 | -0.240 | 1 |  |  |  |  |  |  |
| **Bio17** | 0.667 | -0.638 | -0.438 | 0.062 | 0.694 | 0.650 | -0.382 | 0.634 | 0.638 | 0.711 | 0.603 | 0.817 | 0.679 | 0.990 | -0.640 | 0.685 | 1 |  |  |  |  |  |
| **Bio18** | 0.848 | -0.626 | -0.145 | -0.411 | 0.741 | 0.852 | -0.774 | 0.817 | 0.765 | 0.783 | 0.858 | 0.918 | 0.962 | 0.595 | -0.173 | 0.982 | 0.585 | 1 |  |  |  |  |
| **Bio19** | 0.488 | -0.460 | -0.358 | 0.115 | 0.528 | 0.469 | -0.232 | 0.435 | 0.578 | 0.535 | 0.428 | 0.655 | 0.529 | 0.903 | -0.599 | 0.521 | 0.928 | 0.408 | 1 |  |  |  |
| **Er** | -0.332 | 0.309 | 0.315 | -0.182 | -0.419 | -0.306 | 0.031 | -0.355 | -0.316 | -0.394 | -0.270 | -0.277 | -0.217 | -0.342 | 0.228 | -0.223 | -0.329 | -0.191 | -0.306 | 1 |  |  |
| **Elv** | -0.553 | 0.509 | 0.298 | 0.039 | -0.568 | -0.549 | 0.351 | -0.559 | -0.453 | -0.572 | -0.516 | -0.521 | -0.477 | -0.419 | 0.275 | -0.484 | -0.399 | -0.460 | -0.278 | 0.290 | 1 |  |
| **Gd** | 0.448 | -0.355 | -0.127 | -0.174 | 0.401 | 0.452 | -0.398 | 0.433 | 0.373 | 0.425 | 0.445 | 0.340 | 0.304 | 0.356 | -0.186 | 0.327 | 0.314 | 0.333 | 0.205 | -0.156 | -0.718 | 1 |

Highly correlated (r > 0.7) variables were excluded*.*

**Table 2.2: Correlation among predictor variables from Present.**

|  | **Bio01** | **Bio02** | **Bio03** | **Bio04** | **Bio05** | **Bio06** | **Bio07** | **Bio08** | **Bio09** | **Bio10** | **Bio11** | **Bio12** | **Bio13** | **Bio14** | **Bio15** | **Bio16** | **Bio17** | **Bio18** | **Bio19** | **ER** | **Elv** | **GD** |
| --- | --- | --- | --- | --- | --- | --- | --- | --- | --- | --- | --- | --- | --- | --- | --- | --- | --- | --- | --- | --- | --- | --- |
| **Bio01** | 1 |  |  |  |  |  |  |  |  |  |  |  |  |  |  |  |  |  |  |  |  |  |
| **Bio02** | -0.674 | 1 |  |  |  |  |  |  |  |  |  |  |  |  |  |  |  |  |  |  |  |  |
| **Bio03** | -0.261 | 0.804 | 1 |  |  |  |  |  |  |  |  |  |  |  |  |  |  |  |  |  |  |  |
| **Bio04** | -0.184 | -0.434 | -0.835 | 1 |  |  |  |  |  |  |  |  |  |  |  |  |  |  |  |  |  |  |
| **Bio05** | 0.927 | -0.806 | -0.552 | 0.182 | 1 |  |  |  |  |  |  |  |  |  |  |  |  |  |  |  |  |  |
| **Bio06** | 0.989 | -0.716 | -0.268 | -0.206 | 0.904 | 1 |  |  |  |  |  |  |  |  |  |  |  |  |  |  |  |  |
| **Bio07** | -0.663 | 0.263 | -0.305 | -0.360 | 0.342 | -0.725 | 1 |  |  |  |  |  |  |  |  |  |  |  |  |  |  |  |
| **Bio08** | 0.971 | -0.783 | -0.451 | 0.027 | 0.976 | 0.958 | -0.516 | 1 |  |  |  |  |  |  |  |  |  |  |  |  |  |  |
| **Bio09** | 0.956 | -0.505 | -0.015 | -0.408 | 0.803 | 0.955 | -0.789 | 0.876 | 1 |  |  |  |  |  |  |  |  |  |  |  |  |  |
| **Bio10** | 0.972 | -0.523 | -0.043 | -0.410 | 0.817 | 0.968 | -0.794 | 0.894 | 0.987 | 1 |  |  |  |  |  |  |  |  |  |  |  |  |
| **Bio11** | 0.972 | -0.523 | -0.043 | -0.410 | 0.817 | 0.968 | -0.794 | 0.894 | 0.987 | 0.990 | 1 |  |  |  |  |  |  |  |  |  |  |  |
| **Bio12** | 0.837 | -0.590 | -0.211 | -0.133 | 0.769 | 0.822 | -0.553 | 0.796 | 0.808 | 0.806 | 0.806 | 1 |  |  |  |  |  |  |  |  |  |  |
| **Bio13** | 0.752 | -0.370 | 0.045 | -0.391 | 0.587 | 0.742 | -0.670 | 0.666 | 0.785 | 0.789 | 0.789 | 0.888 | 1 |  |  |  |  |  |  |  |  |  |
| **Bio14** | 0.639 | -0.630 | -0.417 | 0.231 | 0.698 | 0.625 | -0.239 | 0.650 | 0.589 | 0.539 | 0.539 | 0.769 | 0.499 | 1 |  |  |  |  |  |  |  |  |
| **Bio15** | -0.322 | 0.630 | 0.597 | -0.518 | -0.512 | -0.336 | -0.090 | -0.399 | -0.213 | -0.177 | -0.177 | -0.425 | -0.711 | -0.733 | 1 |  |  |  |  |  |  |  |
| **Bio16** | 0.791 | -0.389 | 0.043 | -0.404 | 0.622 | 0.779 | -0.686 | 0.707 | 0.820 | 0.828 | 0.828 | 0.918 | 0.986 | 0.523 | -0.054 | 1 |  |  |  |  |  |  |
| **Bio17** | 0.599 | -0.598 | -0.373 | 0.201 | 0.643 | 0.593 | -0.259 | 0.602 | 0.571 | 0.509 | 0.509 | 0.758 | 0.497 | 0.985 | -0.737 | 0.519 | 1 |  |  |  |  |  |
| **Bio18** | 0.761 | -0.349 | 0.095 | -0.469 | 0.563 | 0.755 | -0.383 | 0.676 | 0.797 | 0.815 | 0.815 | 0.862 | 0.954 | 0.424 | 0.035 | 0.980 | 0.422 | 1 |  |  |  |  |
| **Bio19** | 0.508 | -0.523 | -0.304 | 0.180 | 0.545 | 0.509 | -0.234 | 0.505 | 0.515 | 0.432 | 0.432 | 0.682 | 0.839 | 0.948 | -0.608 | 0.451 | 0.975 | 0.353 | 1 |  |  |  |
| **ER** | 0.008 | 0.000 | -0.010 | -0.001 | 0.009 | 0.003 | 0.008 | 0.004 | -0.004 | 0.007 | 0.007 | 0.009 | -0.004 | 0.006 | 0.001 | 0.000 | -0.007 | -0.006 | -0.016 | 1 |  |  |
| **Elv** | -0.031 | 0.051 | 0.039 | -0.057 | -0.055 | -0.030 | -0.023 | -0.044 | -0.023 | -0.014 | -0.014 | -0.033 | -0.011 | -0.065 | 0.077 | -0.010 | -0.062 | -0.012 | -0.057 | 0.177 | 1 |  |
| **GD** | 0.437 | -0.323 | -0.121 | -0.091 | 0.380 | 0.439 | -0.346 | 0.432 | 0.400 | 0.423 | 0.423 | 0.287 | 0.220 | 0.332 | -0.127 | 0.255 | 0.284 | 0.272 | 0.244 | 0.003 | -0.047 | 1 |

Highly correlated (r > 0.7) variables were excluded*.*

**Table 2.3: Correlation among predictor variables from Future (SSP 1-2.6).**

|  | **Bio01** | **Bio02** | **Bio03** | **Bio04** | **Bio05** | **Bio06** | **Bio07** | **Bio08** | **Bio09** | **Bio10** | **Bio11** | **Bio12** | **Bio13** | **Bio14** | **Bio15** | **Bio16** | **Bio17** | **Bio18** | **Bio19** | **Elv** | **ER** | **GD** |
| --- | --- | --- | --- | --- | --- | --- | --- | --- | --- | --- | --- | --- | --- | --- | --- | --- | --- | --- | --- | --- | --- | --- |
| **Bio01** | 1 |  |  |  |  |  |  |  |  |  |  |  |  |  |  |  |  |  |  |  |  |  |
| **Bio02** | -0.800 | 1 |  |  |  |  |  |  |  |  |  |  |  |  |  |  |  |  |  |  |  |  |
| **Bio03** | -0.477 | 0.827 | 1 |  |  |  |  |  |  |  |  |  |  |  |  |  |  |  |  |  |  |  |
| **Bio04** | -0.174 | -0.234 | -0.728 | 1 |  |  |  |  |  |  |  |  |  |  |  |  |  |  |  |  |  |  |
| **Bio05** | 0.964 | -0.852 | -0.660 | 0.081 | 1 |  |  |  |  |  |  |  |  |  |  |  |  |  |  |  |  |  |
| **Bio06** | 0.990 | -0.849 | -0.499 | -0.182 | 0.950 | 1 |  |  |  |  |  |  |  |  |  |  |  |  |  |  |  |  |
| **Bio07** | -0.719 | 0.565 | 0.008 | 0.655 | -0.537 | -0.773 | 1 |  |  |  |  |  |  |  |  |  |  |  |  |  |  |  |
| **Bio08** | 0.987 | -0.845 | -0.589 | -0.032 | 0.987 | 0.977 | -0.635 | 1 |  |  |  |  |  |  |  |  |  |  |  |  |  |  |
| **Bio09** | 0.982 | -0.742 | -0.349 | -0.321 | 0.908 | 0.979 | -0.804 | 0.947 | 1 |  |  |  |  |  |  |  |  |  |  |  |  |  |
| **Bio10** | 0.979 | -0.866 | -0.636 | 0.028 | 0.995 | 0.970 | -0.702 | 0.996 | 0.932 | 1 |  |  |  |  |  |  |  |  |  |  |  |  |
| **Bio11** | 0.987 | -0.733 | -0.340 | -0.331 | 0.911 | 0.981 | -0.801 | 0.951 | 0.995 | 0.934 | 1 |  |  |  |  |  |  |  |  |  |  |  |
| **Bio12** | 0.868 | -0.703 | -0.411 | -0.183 | 0.821 | 0.857 | -0.650 | 0.844 | 0.862 | 0.840 | 0.860 | 1 |  |  |  |  |  |  |  |  |  |  |
| **Bio13** | 0.798 | -0.558 | -0.183 | -0.392 | 0.692 | 0.790 | -0.729 | 0.754 | 0.825 | 0.728 | 0.827 | 0.918 | 1 |  |  |  |  |  |  |  |  |  |
| **Bio14** | 0.597 | -0.648 | -0.614 | 0.261 | 0.646 | 0.590 | -0.781 | 0.617 | 0.567 | 0.652 | 0.530 | 0.678 | 0.477 | 1 |  |  |  |  |  |  |  |  |
| **Bio15** | -0.507 | 0.726 | 0.733 | -0.396 | -0.608 | -0.534 | 0.807 | -0.550 | -0.448 | -0.597 | -0.424 | -0.566 | -0.258 | -0.709 | 1 |  |  |  |  |  |  |  |
| **Bio16** | 0.808 | -0.538 | -0.163 | -0.413 | 0.700 | 0.793 | -0.722 | 0.758 | 0.834 | 0.732 | 0.839 | 0.939 | 0.985 | 0.472 | -0.281 | 1 |  |  |  |  |  |  |
| **Bio17** | 0.613 | -0.676 | -0.588 | 0.188 | 0.644 | 0.618 | -0.762 | 0.620 | 0.604 | 0.655 | 0.558 | 0.717 | 0.523 | 0.964 | -0.753 | 0.520 | 1 |  |  |  |  |  |
| **Bio18** | 0.754 | -0.482 | -0.094 | -0.470 | 0.625 | 0.741 | -0.736 | 0.705 | 0.782 | 0.766 | 0.795 | 0.862 | 0.941 | 0.735 | -0.775 | 0.958 | 0.784 | 1 |  |  |  |  |
| **Bio19** | 0.526 | -0.600 | -0.518 | 0.160 | 0.547 | 0.536 | -0.336 | 0.529 | 0.537 | 0.561 | 0.480 | 0.639 | 0.461 | 0.934 | -0.702 | 0.452 | 0.978 | 0.713 | 1 |  |  |  |
| **Elv** | -0.052 | 0.056 | 0.065 | -0.041 | -0.063 | -0.050 | 0.010 | -0.060 | -0.047 | -0.061 | -0.043 | -0.037 | -0.016 | -0.072 | 0.065 | -0.017 | -0.059 | -0.010 | -0.050 | 1 |  |  |
| **ER** | -0.017 | 0.023 | 0.008 | 0.004 | -0.014 | -0.022 | 0.031 | -0.019 | -0.024 | -0.016 | -0.017 | -0.006 | -0.019 | -0.008 | 0.012 | -0.012 | -0.023 | -0.019 | -0.028 | 0.201 | 1 |  |
| **GD** | 0.461 | -0.347 | -0.203 | -0.099 | 0.422 | 0.446 | -0.349 | 0.454 | 0.443 | 0.444 | 0.455 | 0.314 | 0.259 | 0.318 | -0.213 | 0.280 | 0.272 | 0.278 | 0.238 | -0.034 | 0.710 | 1 |

Highly correlated (r > 0.7) variables were excluded

**Table 2.4: Correlation among predictor variables from Future (SSP 2-4.5).**

|  | **Bio01** | **Bio02** | **Bio03** | **Bio04** | **Bio05** | **Bio06** | **Bio07** | **Bio08** | **Bio09** | **Bio10** | **Bio11** | **Bio12** | **Bio13** | **Bio14** | **Bio15** | **Bio16** | **Bio17** | **Bio18** | **Bio19** | **Elv** | **ER** | **GD** |
| --- | --- | --- | --- | --- | --- | --- | --- | --- | --- | --- | --- | --- | --- | --- | --- | --- | --- | --- | --- | --- | --- | --- |
| **Bio01** | 1 |  |  |  |  |  |  |  |  |  |  |  |  |  |  |  |  |  |  |  |  |  |
| **Bio02** | -0.853 | 1 |  |  |  |  |  |  |  |  |  |  |  |  |  |  |  |  |  |  |  |  |
| **Bio03** | -0.364 | 0.700 | 1 |  |  |  |  |  |  |  |  |  |  |  |  |  |  |  |  |  |  |  |
| **Bio04** | -0.413 | 0.090 | -0.637 | 1 |  |  |  |  |  |  |  |  |  |  |  |  |  |  |  |  |  |  |
| **Bio05** | 0.969 | -0.882 | -0.549 | -0.183 | 1 |  |  |  |  |  |  |  |  |  |  |  |  |  |  |  |  |  |
| **Bio06** | 0.993 | -0.881 | -0.360 | -0.444 | 0.952 | 1 |  |  |  |  |  |  |  |  |  |  |  |  |  |  |  |  |
| **Bio07** | -0.826 | 0.697 | -0.019 | 0.769 | -0.676 | -0.870 | 1 |  |  |  |  |  |  |  |  |  |  |  |  |  |  |  |
| **Bio08** | 0.987 | -0.883 | -0.477 | -0.286 | 0.989 | 0.976 | -0.753 | 1 |  |  |  |  |  |  |  |  |  |  |  |  |  |  |
| **Bio09** | 0.983 | -0.812 | -0.260 | -0.502 | 0.928 | 0.984 | -0.868 | 0.955 | 1 |  |  |  |  |  |  |  |  |  |  |  |  |  |
| **Bio10** | 0.983 | -0.897 | -0.521 | -0.238 | 0.996 | 0.971 | -0.729 | 0.995 | 0.947 | 1 |  |  |  |  |  |  |  |  |  |  |  |  |
| **Bio11** | 0.989 | -0.805 | -0.236 | -0.541 | 0.924 | 0.989 | -0.887 | 0.957 | 0.989 | 0.946 | 1 |  |  |  |  |  |  |  |  |  |  |  |
| **Bio12** | 0.919 | -0.803 | -0.315 | -0.434 | 0.868 | 0.920 | -0.812 | 0.893 | 0.907 | 0.890 | 0.916 | 1 |  |  |  |  |  |  |  |  |  |  |
| **Bio13** | 0.849 | -0.690 | -0.157 | -0.533 | 0.763 | 0.851 | -0.816 | 0.804 | 0.860 | 0.794 | 0.868 | 0.942 | 1 |  |  |  |  |  |  |  |  |  |
| **Bio14** | 0.760 | -0.793 | -0.606 | 0.000 | 0.807 | 0.750 | -0.503 | 0.768 | 0.736 | 0.808 | 0.702 | 0.805 | 0.707 | 1 |  |  |  |  |  |  |  |  |
| **Bio15** | -0.653 | 0.739 | 0.495 | 0.068 | -0.687 | -0.679 | 0.525 | -0.666 | -0.643 | -0.687 | -0.615 | -0.712 | -0.531 | -0.750 | 1 |  |  |  |  |  |  |  |
| **Bio16** | 0.884 | -0.728 | -0.189 | -0.536 | 0.802 | 0.887 | -0.838 | 0.847 | 0.881 | 0.832 | 0.900 | 0.974 | 0.973 | 0.689 | -0.577 | 1 |  |  |  |  |  |  |
| **Bio17** | 0.713 | -0.767 | -0.532 | -0.063 | 0.737 | 0.719 | -0.541 | 0.707 | 0.722 | 0.746 | 0.670 | 0.777 | 0.696 | 0.960 | -0.778 | 0.664 | 1 |  |  |  |  |  |
| **Bio18** | 0.855 | -0.683 | -0.117 | -0.593 | 0.759 | 0.862 | -0.848 | 0.818 | 0.853 | 0.790 | 0.882 | 0.938 | 0.931 | 0.578 | -0.522 | 0.981 | 0.546 | 1 |  |  |  |  |
| **Bio19** | 0.598 | -0.651 | -0.464 | -0.035 | 0.619 | 0.604 | -0.455 | 0.590 | 0.629 | 0.628 | 0.560 | 0.672 | 0.628 | 0.894 | -0.678 | 0.565 | 0.965 | 0.439 | 1 |  |  |  |
| **Elv** | -0.039 | 0.039 | 0.058 | -0.036 | -0.052 | -0.035 | -0.001 | -0.049 | -0.033 | -0.049 | -0.030 | -0.031 | -0.018 | -0.059 | 0.053 | -0.019 | -0.043 | -0.015 | -0.035 | 1 |  |  |
| **ER** | 0.000 | 0.003 | 0.012 | -0.013 | -0.004 | 0.001 | -0.008 | -0.004 | -0.003 | -0.002 | 0.002 | 0.010 | 0.000 | 0.003 | -0.008 | 0.008 | -0.001 | 0.010 | -0.008 | 0.181 | 1 |  |
| **GD** | 0.527 | -0.437 | -0.171 | -0.230 | 0.499 | 0.526 | -0.460 | 0.522 | 0.513 | 0.518 | 0.525 | 0.438 | 0.400 | 0.395 | -0.306 | 0.416 | 0.344 | 0.398 | 0.299 | -0.027 | 0.721 | 1 |

Highly correlated (r > 0.7) variables were excluded

**Table 2.5: Correlation among predictor variables from Future (SSP 3-7.0).**

|  | **Bio01** | **Bio02** | **Bio03** | **Bio04** | **Bio05** | **Bio06** | **Bio07** | **Bio09** | **Bio10** | **Bio11** | **Bio12** | **Bio13** | **Bio14** | **Bio15** | **Bio16** | **Bio17** | **Bio18** | **Bio19** | **Elv** | **ER** | **GD** |
| --- | --- | --- | --- | --- | --- | --- | --- | --- | --- | --- | --- | --- | --- | --- | --- | --- | --- | --- | --- | --- | --- |
| **Bio01** | 1 |  |  |  |  |  |  |  |  |  |  |  |  |  |  |  |  |  |  |  |  |
| **Bio02** | -0.860 | 1 |  |  |  |  |  |  |  |  |  |  |  |  |  |  |  |  |  |  |  |
| **Bio03** | -0.366 | 0.675 | 1 |  |  |  |  |  |  |  |  |  |  |  |  |  |  |  |  |  |  |
| **Bio04** | -0.731 | 0.426 | -0.200 | 1 |  |  |  |  |  |  |  |  |  |  |  |  |  |  |  |  |  |
| **Bio05** | 0.971 | -0.888 | -0.524 | -0.589 | 1 |  |  |  |  |  |  |  |  |  |  |  |  |  |  |  |  |
| **Bio06** | 0.982 | -0.895 | -0.350 | -0.692 | 0.956 | 1 |  |  |  |  |  |  |  |  |  |  |  |  |  |  |  |
| **Bio07** | -0.843 | 0.763 | 0.043 | 0.732 | -0.742 | -0.906 | 1 |  |  |  |  |  |  |  |  |  |  |  |  |  |  |
| **Bio09** | 0.978 | -0.819 | -0.241 | -0.750 | 0.938 | 0.985 | -0.898 | 1 |  |  |  |  |  |  |  |  |  |  |  |  |  |
| **Bio10** | 0.985 | -0.902 | -0.486 | -0.606 | 0.987 | 0.973 | -0.799 | 0.952 | 1 |  |  |  |  |  |  |  |  |  |  |  |  |
| **Bio11** | 0.992 | -0.819 | -0.282 | -0.809 | 0.943 | 0.970 | -0.856 | 0.977 | 0.957 | 1 |  |  |  |  |  |  |  |  |  |  |  |
| **Bio12** | 0.913 | -0.825 | -0.303 | -0.622 | 0.890 | 0.936 | -0.855 | 0.926 | 0.905 | 0.895 | 1 |  |  |  |  |  |  |  |  |  |  |
| **Bio13** | 0.866 | -0.725 | -0.142 | -0.675 | 0.812 | 0.888 | -0.859 | 0.901 | 0.837 | 0.866 | 0.952 | 1 |  |  |  |  |  |  |  |  |  |
| **Bio14** | 0.808 | -0.844 | -0.568 | -0.396 | 0.854 | 0.823 | -0.649 | 0.796 | 0.844 | 0.768 | 0.847 | 0.768 | 1 |  |  |  |  |  |  |  |  |
| **Bio15** | -0.708 | 0.806 | 0.486 | 0.237 | -0.745 | -0.763 | 0.668 | -0.710 | -0.763 | -0.649 | -0.805 | -0.651 | -0.789 | 1 |  |  |  |  |  |  |  |
| **Bio16** | 0.888 | -0.738 | -0.143 | -0.703 | 0.834 | 0.910 | -0.875 | 0.917 | 0.854 | 0.889 | 0.975 | 0.974 | 0.740 | -0.689 | 1 |  |  |  |  |  |  |
| **Bio17** | 0.755 | -0.814 | -0.497 | -0.354 | 0.786 | 0.791 | -0.674 | 0.765 | 0.793 | 0.716 | 0.820 | 0.770 | 0.965 | -0.798 | 0.714 | 1 |  |  |  |  |  |
| **Bio18** | 0.517 | -0.478 | -0.100 | -0.342 | 0.505 | 0.576 | -0.587 | 0.570 | 0.512 | 0.504 | 0.664 | 0.644 | 0.484 | -0.469 | 0.671 | 0.465 | 1 |  |  |  |  |
| **Bio19** | 0.633 | -0.685 | -0.405 | -0.315 | 0.674 | 0.678 | -0.576 | 0.665 | 0.659 | 0.604 | 0.730 | 0.708 | 0.865 | -0.660 | 0.647 | 0.879 | 0.582 | 1 |  |  |  |
| **Elv** | -0.030 | 0.041 | 0.065 | -0.021 | -0.049 | -0.031 | 0.000 | -0.030 | -0.040 | -0.022 | -0.030 | -0.014 | -0.053 | 0.052 | -0.018 | -0.041 | -0.018 | -0.043 | 1 |  |  |
| **ER** | 0.018 | -0.005 | 0.010 | -0.034 | 0.005 | 0.011 | -0.017 | 0.007 | 0.012 | 0.020 | 0.018 | 0.006 | 0.009 | -0.022 | 0.013 | 0.009 | 0.002 | 0.000 | 0.161 | 1 |  |
| **GD** | 0.594 | -0.501 | -0.179 | -0.446 | 0.558 | 0.589 | -0.541 | 0.584 | 0.580 | 0.589 | 0.523 | 0.487 | 0.486 | -0.425 | 0.504 | 0.436 | 0.287 | 0.299 | -0.019 | 0.741 | 1 |

Highly correlated (r > 0.7) variables were excluded

**Table 2.6: Correlation among predictor variables from Future (SSP 5-8.5).**

|  | **Bio01** | **Bio02** | **Bio03** | **Bio04** | **Bio05** | **Bio06** | **Bio07** | **Bio08** | **Bio09** | **Bio10** | **Bio11** | **Bio12** | **Bio13** | **Bio14** | **Bio15** | **Bio16** | **Bio17** | **Bio18** | **Bio19** | **Elv** | **ER** | **GD** |
| --- | --- | --- | --- | --- | --- | --- | --- | --- | --- | --- | --- | --- | --- | --- | --- | --- | --- | --- | --- | --- | --- | --- |
| **Bio01** | 1 |  |  |  |  |  |  |  |  |  |  |  |  |  |  |  |  |  |  |  |  |  |
| **Bio02** | -0.881 | 1 |  |  |  |  |  |  |  |  |  |  |  |  |  |  |  |  |  |  |  |  |
| **Bio03** | -0.312 | 0.616 | 1 |  |  |  |  |  |  |  |  |  |  |  |  |  |  |  |  |  |  |  |
| **Bio04** | -0.496 | 0.224 | -0.613 | 1 |  |  |  |  |  |  |  |  |  |  |  |  |  |  |  |  |  |  |
| **Bio05** | 0.952 | -0.907 | -0.560 | -0.813 | 1 |  |  |  |  |  |  |  |  |  |  |  |  |  |  |  |  |  |
| **Bio06** | 0.993 | -0.908 | -0.308 | -0.721 | 0.936 | 1 |  |  |  |  |  |  |  |  |  |  |  |  |  |  |  |  |
| **Bio07** | -0.827 | 0.703 | -0.121 | 0.844 | -0.733 | -0.864 | 1 |  |  |  |  |  |  |  |  |  |  |  |  |  |  |  |
| **Bio08** | 0.987 | -0.904 | -0.435 | -0.736 | 0.983 | 0.975 | -0.742 | 1 |  |  |  |  |  |  |  |  |  |  |  |  |  |  |
| **Bio09** | 0.980 | -0.832 | -0.190 | -0.859 | 0.900 | 0.981 | -0.874 | 0.950 | 1 |  |  |  |  |  |  |  |  |  |  |  |  |  |
| **Bio10** | 0.981 | -0.916 | -0.478 | -0.921 | 0.992 | 0.970 | -0.719 | 0.996 | 0.941 | 1 |  |  |  |  |  |  |  |  |  |  |  |  |
| **Bio11** | 0.990 | -0.842 | -0.190 | -0.910 | 0.902 | 0.989 | -0.890 | 0.957 | 0.989 | 0.946 | 1 |  |  |  |  |  |  |  |  |  |  |  |
| **Bio12** | 0.933 | -0.838 | -0.264 | -0.718 | 0.862 | 0.932 | -0.822 | 0.905 | 0.915 | 0.902 | 0.932 | 1 |  |  |  |  |  |  |  |  |  |  |
| **Bio13** | 0.893 | -0.772 | -0.139 | -0.761 | 0.784 | 0.897 | -0.856 | 0.853 | 0.894 | 0.840 | 0.910 | 0.957 | 1 |  |  |  |  |  |  |  |  |  |
| **Bio14** | 0.824 | -0.849 | -0.554 | -0.861 | 0.862 | 0.822 | -0.578 | 0.837 | 0.797 | 0.865 | 0.781 | 0.844 | 0.766 | 1 |  |  |  |  |  |  |  |  |
| **Bio15** | -0.716 | 0.771 | 0.437 | 0.986 | -0.738 | -0.738 | 0.570 | -0.727 | -0.703 | -0.739 | -0.686 | -0.785 | -0.647 | -0.789 | 1 |  |  |  |  |  |  |  |
| **Bio16** | 0.918 | -0.779 | -0.142 | -0.713 | 0.810 | 0.915 | -0.858 | 0.877 | 0.909 | 0.864 | 0.932 | 0.982 | 0.981 | 0.763 | -0.683 | 1 |  |  |  |  |  |  |
| **Bio17** | 0.779 | -0.820 | -0.496 | -0.792 | 0.802 | 0.789 | -0.592 | 0.780 | 0.774 | 0.811 | 0.746 | 0.821 | 0.752 | 0.970 | -0.804 | 0.740 | 1 |  |  |  |  |  |
| **Bio18** | 0.828 | -0.668 | 0.020 | -0.603 | 0.380 | 0.830 | -0.859 | 0.780 | 0.825 | 0.745 | 0.862 | 0.887 | 0.906 | 0.946 | -0.775 | 0.931 | 0.510 | 1 |  |  |  |  |
| **Bio19** | 0.665 | -0.719 | -0.414 | -0.187 | 0.676 | 0.685 | -0.544 | 0.657 | 0.688 | 0.689 | 0.642 | 0.723 | 0.678 | 0.912 | -0.745 | 0.642 | 0.967 | 0.404 | 1 |  |  |  |
| **Elv** | -0.034 | 0.037 | 0.062 | -0.030 | -0.050 | -0.029 | -0.007 | -0.042 | -0.027 | -0.043 | -0.025 | -0.030 | -0.018 | -0.052 | 0.044 | -0.024 | -0.041 | -0.001 | -0.031 | 1 |  |  |
| **ER** | -0.004 | 0.008 | 0.012 | -0.009 | -0.007 | -0.004 | -0.002 | -0.008 | -0.007 | -0.007 | -0.003 | 0.005 | -0.002 | -0.002 | 0.001 | 0.003 | -0.003 | 0.007 | -0.006 | 0.162 | 1 |  |
| **GD** | 0.579 | -0.491 | -0.163 | -0.307 | 0.540 | 0.573 | -0.492 | 0.563 | 0.559 | 0.565 | 0.576 | 0.507 | 0.510 | 0.481 | -0.342 | 0.522 | 0.435 | 0.436 | 0.361 | -0.020 | 0.732 | 1 |

Highly correlated (r > 0.7) variables were excluded
